# Inhibition of myocardial cathepsin-L release during reperfusion following myocardial infarction improves cardiac function and reduces infarct size

**DOI:** 10.1093/cvr/cvab204

**Published:** 2021-06-16

**Authors:** Weihong He, Charlotte S McCarroll, Katrin Nather, Kristopher Ford, Kenneth Mangion, Alexandra Riddell, Dylan O’Toole, Ali Zaeri, David Corcoran, David Carrick, Mathew M Y Lee, Margaret McEntegart, Andrew Davie, Richard Good, Mitchell M Lindsay, Hany Eteiba, Paul Rocchiccioli, Stuart Watkins, Stuart Hood, Aadil Shaukat, Lisa McArthur, Elspeth B Elliott, John McClure, Catherine Hawksby, Tamara Martin, Mark C Petrie, Keith G Oldroyd, Godfrey L Smith, Oxford Acute Myocardial Infarction (OxAMI) Study, Keith M Channon, Colin Berry, Stuart A Nicklin, Christopher M Loughrey

**Affiliations:** Institute of Cardiovascular and Medical Sciences, College of Medical, Veterinary and Life Sciences, Glasgow Cardiovascular Research Centre, University of Glasgow, University Place, Glasgow G12 8TA, UK; Institute of Cardiovascular and Medical Sciences, College of Medical, Veterinary and Life Sciences, Glasgow Cardiovascular Research Centre, University of Glasgow, University Place, Glasgow G12 8TA, UK; Institute of Cardiovascular and Medical Sciences, College of Medical, Veterinary and Life Sciences, Glasgow Cardiovascular Research Centre, University of Glasgow, University Place, Glasgow G12 8TA, UK; Institute of Cardiovascular and Medical Sciences, College of Medical, Veterinary and Life Sciences, Glasgow Cardiovascular Research Centre, University of Glasgow, University Place, Glasgow G12 8TA, UK; Institute of Cardiovascular and Medical Sciences, College of Medical, Veterinary and Life Sciences, Glasgow Cardiovascular Research Centre, University of Glasgow, University Place, Glasgow G12 8TA, UK; West of Scotland Heart and Lung Centre, Golden Jubilee National Hospital, Clydebank G81 4DY, UK; Institute of Cardiovascular and Medical Sciences, College of Medical, Veterinary and Life Sciences, Glasgow Cardiovascular Research Centre, University of Glasgow, University Place, Glasgow G12 8TA, UK; Institute of Cardiovascular and Medical Sciences, College of Medical, Veterinary and Life Sciences, Glasgow Cardiovascular Research Centre, University of Glasgow, University Place, Glasgow G12 8TA, UK; Institute of Cardiovascular and Medical Sciences, College of Medical, Veterinary and Life Sciences, Glasgow Cardiovascular Research Centre, University of Glasgow, University Place, Glasgow G12 8TA, UK; Institute of Cardiovascular and Medical Sciences, College of Medical, Veterinary and Life Sciences, Glasgow Cardiovascular Research Centre, University of Glasgow, University Place, Glasgow G12 8TA, UK; West of Scotland Heart and Lung Centre, Golden Jubilee National Hospital, Clydebank G81 4DY, UK; Institute of Cardiovascular and Medical Sciences, College of Medical, Veterinary and Life Sciences, Glasgow Cardiovascular Research Centre, University of Glasgow, University Place, Glasgow G12 8TA, UK; West of Scotland Heart and Lung Centre, Golden Jubilee National Hospital, Clydebank G81 4DY, UK; Institute of Cardiovascular and Medical Sciences, College of Medical, Veterinary and Life Sciences, Glasgow Cardiovascular Research Centre, University of Glasgow, University Place, Glasgow G12 8TA, UK; West of Scotland Heart and Lung Centre, Golden Jubilee National Hospital, Clydebank G81 4DY, UK; West of Scotland Heart and Lung Centre, Golden Jubilee National Hospital, Clydebank G81 4DY, UK; West of Scotland Heart and Lung Centre, Golden Jubilee National Hospital, Clydebank G81 4DY, UK; West of Scotland Heart and Lung Centre, Golden Jubilee National Hospital, Clydebank G81 4DY, UK; West of Scotland Heart and Lung Centre, Golden Jubilee National Hospital, Clydebank G81 4DY, UK; West of Scotland Heart and Lung Centre, Golden Jubilee National Hospital, Clydebank G81 4DY, UK; West of Scotland Heart and Lung Centre, Golden Jubilee National Hospital, Clydebank G81 4DY, UK; West of Scotland Heart and Lung Centre, Golden Jubilee National Hospital, Clydebank G81 4DY, UK; West of Scotland Heart and Lung Centre, Golden Jubilee National Hospital, Clydebank G81 4DY, UK; West of Scotland Heart and Lung Centre, Golden Jubilee National Hospital, Clydebank G81 4DY, UK; Institute of Cardiovascular and Medical Sciences, College of Medical, Veterinary and Life Sciences, Glasgow Cardiovascular Research Centre, University of Glasgow, University Place, Glasgow G12 8TA, UK; Institute of Cardiovascular and Medical Sciences, College of Medical, Veterinary and Life Sciences, Glasgow Cardiovascular Research Centre, University of Glasgow, University Place, Glasgow G12 8TA, UK; Institute of Cardiovascular and Medical Sciences, College of Medical, Veterinary and Life Sciences, Glasgow Cardiovascular Research Centre, University of Glasgow, University Place, Glasgow G12 8TA, UK; Institute of Cardiovascular and Medical Sciences, College of Medical, Veterinary and Life Sciences, Glasgow Cardiovascular Research Centre, University of Glasgow, University Place, Glasgow G12 8TA, UK; Institute of Cardiovascular and Medical Sciences, College of Medical, Veterinary and Life Sciences, Glasgow Cardiovascular Research Centre, University of Glasgow, University Place, Glasgow G12 8TA, UK; Institute of Cardiovascular and Medical Sciences, College of Medical, Veterinary and Life Sciences, Glasgow Cardiovascular Research Centre, University of Glasgow, University Place, Glasgow G12 8TA, UK; West of Scotland Heart and Lung Centre, Golden Jubilee National Hospital, Clydebank G81 4DY, UK; West of Scotland Heart and Lung Centre, Golden Jubilee National Hospital, Clydebank G81 4DY, UK; Institute of Cardiovascular and Medical Sciences, College of Medical, Veterinary and Life Sciences, Glasgow Cardiovascular Research Centre, University of Glasgow, University Place, Glasgow G12 8TA, UK; NIHR Oxford Biomedical Research Centre, John Radcliffe Hospital, Oxford, UK; Division of Cardiovascular Medicine, British Heart Foundation Centre of Research Excellence, University of Oxford, Oxford, UK; Institute of Cardiovascular and Medical Sciences, College of Medical, Veterinary and Life Sciences, Glasgow Cardiovascular Research Centre, University of Glasgow, University Place, Glasgow G12 8TA, UK; West of Scotland Heart and Lung Centre, Golden Jubilee National Hospital, Clydebank G81 4DY, UK; Institute of Cardiovascular and Medical Sciences, College of Medical, Veterinary and Life Sciences, Glasgow Cardiovascular Research Centre, University of Glasgow, University Place, Glasgow G12 8TA, UK; Institute of Cardiovascular and Medical Sciences, College of Medical, Veterinary and Life Sciences, Glasgow Cardiovascular Research Centre, University of Glasgow, University Place, Glasgow G12 8TA, UK

**Keywords:** Myocardial infarction, Reperfusion injury, Cardiomyocytes, Calcium, Sarcoplasmic reticulum

## Abstract

**Aims:**

Identifying novel mediators of lethal myocardial reperfusion injury that can be targeted during primary percutaneous coronary intervention (PPCI) is key to limiting the progression of patients with ST-elevation myocardial infarction (STEMI) to heart failure. Here, we show through parallel clinical and integrative preclinical studies the significance of the protease cathepsin-L on cardiac function during reperfusion injury.

**Methods and results:**

We found that direct cardiac release of cathepsin-L in STEMI patients (*n* = 76) immediately post-PPCI leads to elevated serum cathepsin-L levels and that serum levels of cathepsin-L in the first 24 h post-reperfusion are associated with reduced cardiac contractile function and increased infarct size. Preclinical studies demonstrate that inhibition of cathepsin-L release following reperfusion injury with CAA0225 reduces infarct size and improves cardiac contractile function by limiting abnormal cardiomyocyte calcium handling and apoptosis.

**Conclusion:**

Our findings suggest that cathepsin-L is a novel therapeutic target that could be exploited clinically to counteract the deleterious effects of acute reperfusion injury after an acute STEMI.

## 1. Introduction

Acute coronary artery occlusion leading to ST-elevation myocardial infarction (STEMI) is a major cause of heart failure and death globally. Primary percutaneous coronary intervention (PPCI) is the most effective treatment for restoring blood flow to ischaemic myocardium and limiting cardiomyocyte death following an acute STEMI.^1^ Nonetheless, the effectiveness of PPCI is substantially reduced following activation of mediators that paradoxically increase susceptibility to cardiomyocyte death by disrupting normal calcium handling.^2^ Ultimately, this lethal myocardial reperfusion injury contributes to up to 50% of the final infarct size^2^ (a major determinate of clinical outcome); reduced contractility; and adverse cardiac remodelling post-STEMI, which can lead to heart failure.^3^^,^^4^ The latter is particularly important given its association with substantial mortality, poor quality of life and appreciable health-economic burden.^5^ Novel therapeutic targets with the potential to reduce the impact of reperfusion injury and adverse cardiac remodelling are therefore urgently required to treat patients with STEMI and prevent progression to heart failure.^6^

Cathepsins are proteases classified by structure and catalytic type into serine, aspartic and cysteine cathepsins. These enzymes were traditionally thought to be located exclusively within intracellular lysosomes and only play a role in homeostatic protein breakdown.^7^ However, cathepsins are now known to be important signalling molecules involved in numerous vital cellular processes such as remodelling and differentiation.^7^ Cathepsins are secreted into the extracellular space in large amounts by fusion of lysosomal organelles with the plasma membrane and/or exocytosis.^7^ Serum levels of cathepsin-L (a cysteine cathepsin) are elevated among patients with coronary artery stenosis and coronary heart disease (CHD).^8–10^ Nevertheless, it is unknown whether increased capthepsin-L simply reflects cell death or if it has a biological function, particularly in the clinically relevant context of reperfusion injury.

Our integrative clinical and experimental study reveals the vital role and clinical relevance for cathepsin-L during myocardial reperfusion injury in rodents and humans.

## 2. Methods

A detailed description of the methods and statistical analysis is reported in Supplementary material online.

### 2.1 Human studies

A prospective single-centre cohort study was performed at a regional cardiac centre (Golden Jubilee National Hospital, Scotland, UK). Research staff screened patients with acute STEMI undergoing emergency invasive management. In all, 83 patients agreed to participate and provided written informed consent. The human studies conformed to the principles outlined in the Declaration of Helsinki. Systemic blood samples were taken at pre-reperfusion of the occluded culprit coronary artery; 20 min post-PPCI; 24 h post-PPCI; and at 6 months post-PPCI. Cardiac magnetic resonance imaging (MRI) was performed at 24 h and 6 months post-PPCI (60 patients used for area under the curve analysis; Supplementary material online, *Figure* *S1*). The study protocol and consent processes were approved by the local Research Ethics Committee (REC 14/WS/0085).

In a separate study, patients undergoing emergency PPCI for STEMI at the John Radcliffe Hospital, Oxford, UK were recruited as part of the Oxford Acute Myocardial Infarction (OxAMI) study. Assent for participation was obtained at the time of PPCI. Blood samples were obtained at the end of the PPCI procedure from the coronary artery guide catheter, from a catheter placed in the coronary sinus, and from a peripheral vein.^11^ Informed written consent for continued participation was obtained within the following 24 h. The study protocol and consent processes were approved by the local Research Ethics Committee (REC 11/SC/0397).

Serum levels of cathepsin-L were measured by enzyme-linked immunosorbent assay using the manufacturer’s recommended protocol (USCN Life Science Inc.) and normalized to the total serum protein content.

### 2.2 Experimental studies

The care and use of animals were in accordance with the UK Government Animals (Scientific Procedures) Act 1986. All animal procedures (including euthanasia by schedule one cervical dislocation) were approved by the University of Glasgow Animal Welfare and Ethical Review Body and licensed by the Home Office, UK (project license numbers 600/4503 and P06FE1F82).

Left intraventricular pressure was measured in Langendorff perfused hearts from adult male Wistar rats. Infarct size was measured using triphenyltetrazolium chloride staining. Cathepsin-L activity was determined using established protocols. The cathepsin-L inhibitor CAA0225 (Calbiochem)^12^ was prepared in DMSO (dimethyl sulfoxide).

Adult rat cardiomyocytes were isolated as previously described^13^ and used for epifluoresent and confocal calcium measurements.

The *in vivo* model of reperfusion injury involved male C57Bl/6 mice aged 9–12 weeks. These mice were anaesthetised in an induction chamber with 4% isoflurane and 100% oxygen. The anaesthetised mice were intubated and ventilated using a small animal respirator (Harvard Apparatus, Germany) and maintained with 1.5% isoflurane and underwent temporary coronary artery ligation via thoracotomy.

## 3. Results

### 3.1 Serum cathepsin-L and MRI parameters among STEMI patients

In all, 83 STEMI patients agreed to participate in the current study (Supplementary material online, *Figure S1*). The demographic characteristics are shown in Supplementary material online, *Table S1*.

**Figure 1:**
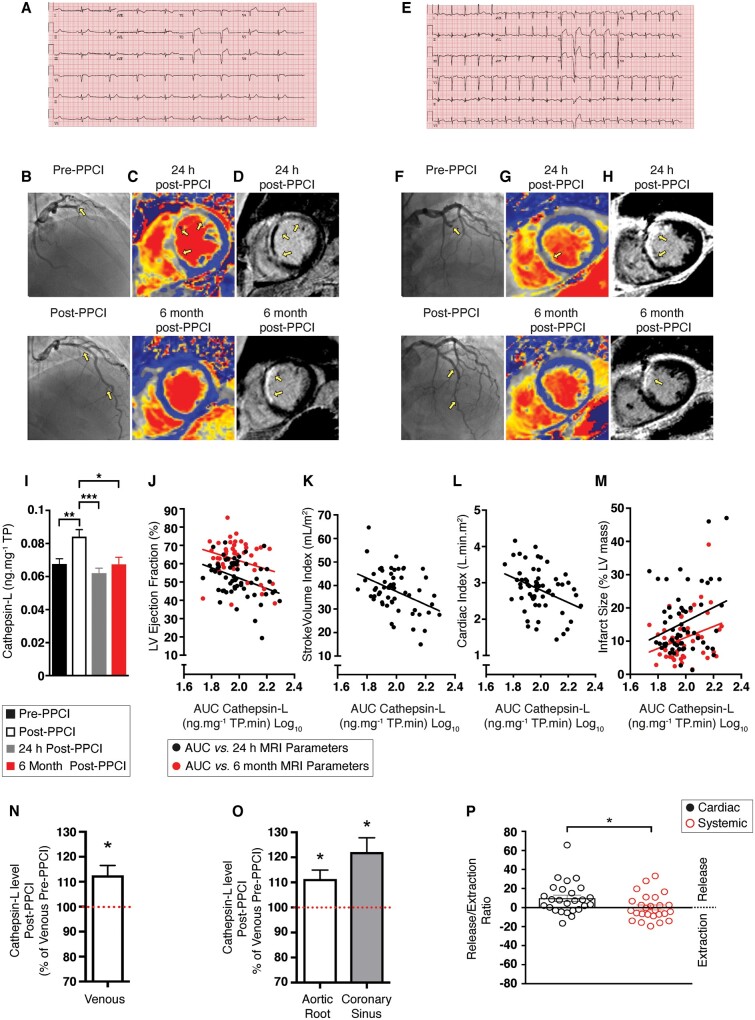
Cathepsin-L levels among STEMI patients with divergent outcomes. Full details for this figure are provided elsewhere (Supplementary material online, *Figure S1* extended legend). (*A*–*D*) Data for a patient with high level of cathepsin [2.12 ng.mg^−1^ total protein (TP) Log_10_]. (*A*) Pre-PPCI ECG. (*B*) Angiography pre-PPCI (top) and post-PPI (bottom). The yellow arrows indicate restoration of TIMI 3 flow. (*C*) Cardiac MRI performed pre-PPCI (top) and post-PPI (bottom) showing remote myocardium (blue) and ischaemic myocardium (grey; yellow arrows). (*D*) Infarct size pre-PPCI (top) and post-PPI (bottom) showing normal myocardium nulled to black; acute scar depicted as hyperenhanced (white); and a central hypoenhanced region representing microvascular obstruction. (*E*–*H*) Data from a patient with a low level of cathepsin (1.84 ng.mg^−1^ TP Log_10_). (*E*) Pre-PPCI ECG. (*F*) Angiography pre-PPCI (top) and post-PPI (bottom). (*G*) Cardiac MRI performed pre-PPCI (top) and post-PPI (bottom). (*H*) Infarct size pre-PPCI (top) and post-PPI (bottom). (*I*) Serum cathepsin-L levels among patients undergoing PPCI. (*J*–*L*) Correlations (*P *<* *0.05) between MRI parameters at 24 h post-PPCI (black) and 6 months post-PPCI (red) and the area under the curve (AUC) of cathepsin-L levels measured in the first 24 h post-MI. (*J*) LV ejection fraction. (*K*) Stroke volume indexed for body surface area (right). (*L*) Cardiac index. (*M*) Infarct size (see Supplementary material online, *Figure S1* for *n* values for *I*–*M*). (*N*–*P*) Cathepsin-L levels among STEMI patients immediately after primary PCI (*n* = 26). Cathepsin-L levels in (*N*) Venous (*O*) Aortic root and coronary sinus blood samples, allowing determination of (*P*) Cardiac and systemic release (above 0) and extraction (below 0) ratios. Data were assessed for normality and are expressed as mean ± standard error of the mean (SEM). Statistical comparisons were made by a two-sample Student’s *T*-test on the raw data. Multiple groups were compared with analysis of variance (ANOVA). A significance level of *P *<* *0.05 was considered significant. Pearson correlation was used to investigate the association between log_10_ cathepsin levels and subsequent MRI parameters in patients undergoing PPCI.

Angiography and electrocardiogram conducted pre-PPCI and post-PPCI confirmed the presence of STEMI (*Figure 1A, B, E, and F*). Infarct size and left ventricular (LV) function were assessed by MRI at 24 h and 6 months post-PPCI (*Figure 1C, D, G, and H*). Serum cathepsin-L levels increased 20 min post-PPCI to 135% of pre-PPCI levels (*Figure 1I*;  *P *<* *0.05). Area under the curve analyses indicated that the 24-h log_10_ cathepsin-L levels were negatively correlated with contractile parameters measured by MRI at 24 h post-PPCI (*Figure 1J, K, and L*): LV ejection fraction (LVEF; *r* = –0.36, *P *=* *0.004); stroke volume indexed to body surface area (*r* = –0.41, *P *=* *0.001); and cardiac index (*r* = –0.36, *P *=* *0.005). The LVEF at 6 months, negatively correlated with cathepsin-L levels at baseline (*r* = –0.29, *P *=* *0.049; *Figure 1J*), which suggested prognostic significance of baseline serum cathepsin-L concentrations for long-term LV function. Elevated cathepsin-L levels were also associated with increased infarct sizes at 24 h (*r* = 0.28, *P *=* *0.032) and 6 months (*r* = 0.30, *P *=* *0.032; *Figure 1M*).

Patients received appropriate pharmacological management post-PPCI (Supplementary material online, *Table S2*) and mean cardiac performance improved over time post-PPCI (Supplementary material online, *Figure S2*).

### 3.2 Cardiac release of cathepsin-L post-PPCI

Serum cathepsin-L levels were also assessed pre-PPCI and immediately post-PPCI in a cohort of STEMI patients treated at a different cardiac care centre (*n* = 26). The post-PPCI levels were 113% of the pre-PPCI levels (*P *<* *0.05; *Figure 1N*), thus demonstrating the robustness of these findings across two geographically distinct locations. Placement of catheters in the second cohort enabled parallel collection of aortic root, coronary sinus and venous blood samples post-PPCI. Cathepsin-L levels in aortic root and coronary sinus post-PPCI were higher than those detected in venous blood samples taken before PPCI (111% and 122% of pre-PPCI levels, respectively *P *<* *0.05; *Figure 1O*). Furthermore, post-PPCI serum cathepsin-L levels in coronary sinus samples were higher than those found in post-PPCI aortic root samples, resulting in a mean cardiac release ratio of 9.8 ± 3.3 (*P *<* *0.05; *Figure 1P*, left). In contrast, serum cathepsin-L levels in post-PPCI venous blood and aortic root samples were similar (mean systemic release ratio 0.1 ± 2.6, *P *>* *0.05; *Figure 1P*, right).

### 3.3 Cardiac function in IR mouse hearts treated with CAA0225 *in vivo*

**Figure 2:**
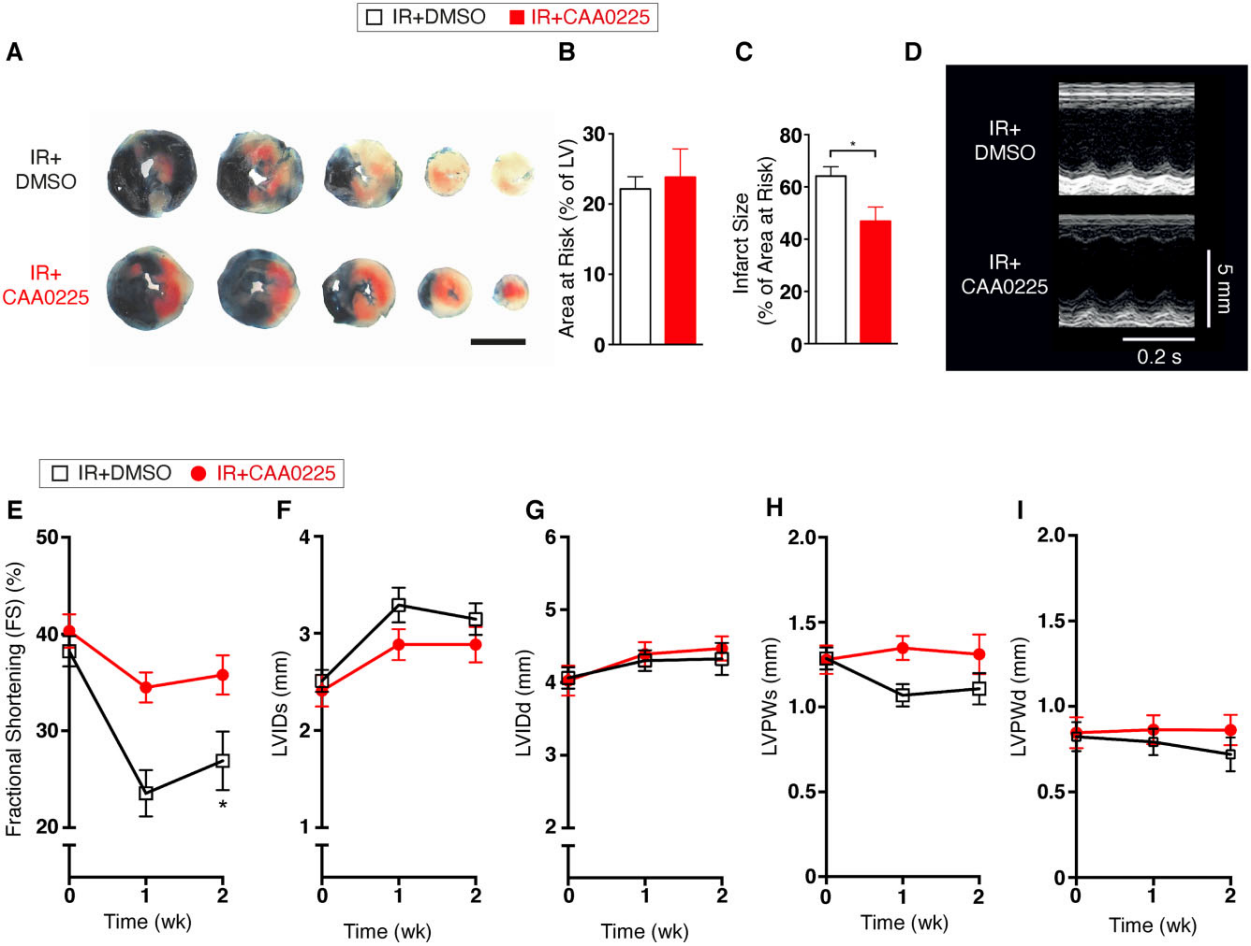
Cardiac function in reperfused mouse hearts treated with the cathepsin-L inhibitor CAA0225 *in vivo*. Data were collected at 2 weeks after reperfusion. (*A*) Typical Evans blue and TTC staining to delineate infarct size and area at risk (3 mm scale bar). (*B*) Mean area at risk for IR+DMSO (*n* = 13) and IR+ CAA0225 (*n* = 14). (*C*) Mean infarct size. (*D*) Typical M-mode echocardiographic images of IR+DMSO and IR+CAA0225 at 0 week and 2 weeks post-reperfusion. (*E*–*I*) Mean echocardiographic data for IR+DMSO (*n* = 7) and IR+CAA0225 (*n* = 8). (*E*) Fractional shortening (FS). (*F*) Left ventricular internal diameter at systole (LVIDs). (*G*) LVID at diastole (LVIDd). (*H*) LV posterior wall thickness at diastole (LVPWd). (*I*) LVPW thickness at systole (LVPWs). * *P *<* *0.05. Data were assessed for normality and are expressed as mean ± standard error of the mean (SEM). Statistical comparisons were made by a two-sample Student’s *T*-test on the raw data. Multiple groups were compared with analysis of variance (ANOVA). A significance level of *P *<* *0.05 was considered significant. The data were analysed using analysis between groups at a particular time point (where significance is shown it depicts difference between groups; not from Time 0).

Given the findings that elevated levels of cathepsin-L were associated with larger infarct size and reduced cardiac function among STEMI patients, we next sought to determine whether blocking cathepsin-L activity using a specific cathepsin-L inhibitor (CAA0225)^12^ improves cardiac function using an animal model of myocardial reperfusion injury *in vivo*. No statistically significant difference was found in the area at risk of infarction for the CAA0225 group [ischaemia reperfusion (IR)+CAA0225] vs. the control group (IR+DMSO; *Figure 2A and B*), demonstrating that the coronary artery ligation placement was equivalent. However, the infarct size among mice treated with CAA0025 was 73% of that observed among control mice (*Figure 2A and C*). M-mode echocardiography was performed before and after IR to assess cardiac contractile function (*Figure 2D*). Fractional shortening was decreased in the control group over the 2-week period, whereas CAA0225-treated mice exhibited preserved fractional shortening (*Figure 2E*). No statistically significant between-group differences were found for LV internal dimension at systole (*Figure 2F*) or diastole (*Figure 2G*). Similarly, LV posterior wall thickness at systole (*Figure 2H*) and diastole (*Figure 2I*) did not differ between the two groups.

**Figure 3:**
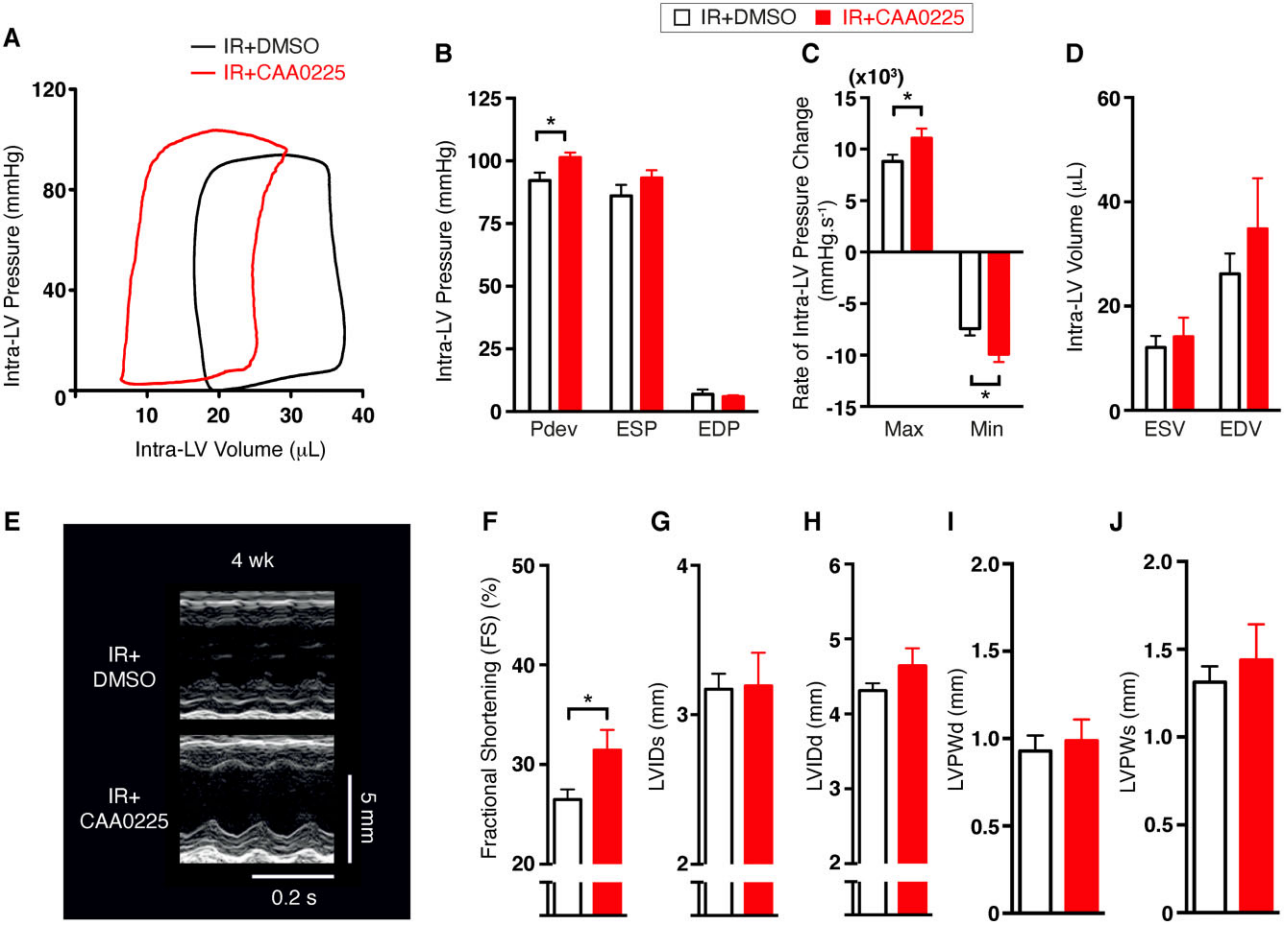
Haemodynamic and cardiac function in reperfused mouse hearts treated with the cathepsin-L inhibitor CAA0225 *in vivo*. Data were collected at 2 weeks and 4 weeks after reperfusion. (*A*) Typical pressure volume (PV) loops. (*B*) Mean PV loop intra-LV pressure measurements for IR+ DMSO (*n* = 5) and IR = CAA0225 (*n* = 4). (*C*) Mean rate of intra-LV pressure change. (*D*) Mean intra-LV volume. (*E*) Typical M-mode echocardiographic images of IR+DMSO and IR+CAA0225 at 4 weeks post-reperfusion. (*F*–*J*) Mean echocardiographic data at 4 weeks post-reperfusion for IR+DMSO (*n* = 7) and IR+CAA0225 (*n* = 8). (*F*) Fractional shortening (FS). (*G*) LV internal diameter at systole (LVIDs). (*H*) LVID at diastole (LVIDd). (*I*) LV posterior wall thickness at diastole (LVPWd). (*J*) LVPW thickness at systole (LVPWs). * *P *<* *0.05. Data were assessed for normality and are expressed as mean ± standard error of the mean (SEM). Statistical comparisons were made by a two-sample Student’s *T*-test on the raw data. Multiple groups were compared with analysis of variance (ANOVA). A significance level of *P *<* *0.05 was considered significant.

Pressure–volume (PV) loop measurements were also performed at 2 weeks to assess hemodynamic function (*Figure 3A*). Compared with the control mice, CAA0225-treated mice demonstrated increased intra-LV pressure parameters, including developed pressure (*Figure 3B*;  *P *<* *0.05), maximum rate of intra-LV pressure rise (*Figure 3C*), and minimum rate of intra-LV pressure fall (*Figure 3C*; *P *<* *0.05). No statistically significant between-group difference was found for end-systolic or diastolic pressure (*Figure 3B*) or for end-systolic or diastolic volume (*Figure 3D*).

A separate cohort of mice was assessed to establish whether the beneficial effects observed at 2-week post-IR injury were maintained at 4 weeks (*Figure 3E*). Fractional shortening was preserved in CAA0225-treated vs. control mice (*Figure 3F*;  *P *<* *0.05); however, no statistically significant differences were found for LV internal diameter at systole or diastole, and wall thickness at diastole or systole (*Figure 3G*–*J*). These data support our hypothesis that therapeutic targeting of cathepsin-L with a specific inhibitor (CAA0225) can reduce infarct size and preserve cardiac contractile function post-reperfusion injury.

### 3.4 Cardiac function in *ex vivo* rat hearts during reperfusion injury pretreated with CAA0225

**Figure 4:**
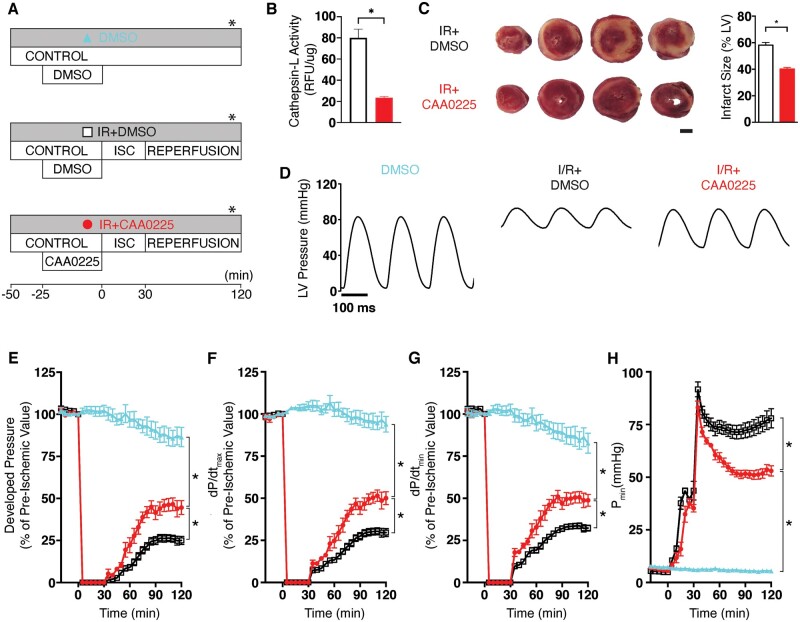
Cardiac function in *ex vivo* rat hearts during IR injury pretreated with the cathepsin-L inhibitor CAA0225. (*A*) Schematic of the three protocols used. (*B*) Cathepsin-L activity measured in left ventricular tissue IR+DMSO (white bar; *n* = 5) and IR+CAA0225 (red bar; *n* = 5). (*C*) Typical TTC staining of heart slices, where the red staining represents live tissue and the pale unstained colour is dead tissue (3 mm scale bar). The mean infarct size is shown on the right for IR+DMSO (*n* = 11) and IR+CAA0225 (*n* = 6). (*D*) Typical LV pressure measured at the time point shown in part A of this figure (*). (*E*–H) Mean LV pressure data for DMSO (*n* = 6), IR+DMSO (*n* = 15) and IR+CAA0225 (*n* = 6). (*E*) Developed LV pressure. (*F*) Maximum rate of rise (dP/dt_max_). (*G*) Maximum rate of fall (dP/dt_min_). (*H*) Minimum (P_min_). *P <* *0.05. Data were assessed for normality and are expressed as mean ± standard error of the mean (SEM). Statistical comparisons were made by a two-sample Student’s *T*-test on the raw data. Multiple groups were compared with analysis of variance (ANOVA). A significance level of *P *<* *0.05 was considered significant. In experiments involving serial measurements on *ex vivo* hearts the final measurement was taken as the relevant summary statistic in order to answer the research hypothesis of whether there would be changes in population mean values between treatment groups by the end of the experiment.

To determine the direct effects of CAA0225 on cardiac contractile function, Langendorff-perfused *ex vivo* whole rat hearts were treated as shown in *Figure 4A*. Cathepsin-L activity in CAA0225-treated IR hearts was reduced to 29% of that in the IR+DMSO group (*Figure 4B*). CAA0225 decreased infarct size to 69% of that in the IR+DSMO group (*Figure 4C*). CAA0225 also improved overall LV developed pressure post-reperfusion (*Figure 4D*). The P_dev_ in CAA0225-treated hearts at 120 min post-reperfusion was 178% of that in the IR+DMSO group (*Figure 4E*). In the IR+CAA0225 group, the dP/dt_max_ and dP/dt_min_ (measures of systolic and diastolic function, respectively) at 120 min were 169% and 151% of those in the IR+DMSO group, respectively (*Figure 4F and G*). No statistically significant change was found between the IR+CAA0225 and IR+DMSO groups for P_min_ (a measure of the ability of the heart to relax) during the ischaemic period. However, this measure was reduced during reperfusion with CAA0225 (53 ± 2 mmHg), which was 68% of that in the IR+DMSO group at 120 min (78 ± 5 mmHg; *Figure 4H*;  *P *<* *0.05). CAA0225 therefore reduced infarct size and improved the systolic and diastolic contractile function of *ex vivo* rat hearts post-reperfusion injury.

### 3.5 Effect of CAA0225 applied during reperfusion on cardiac function

**Figure 5:**
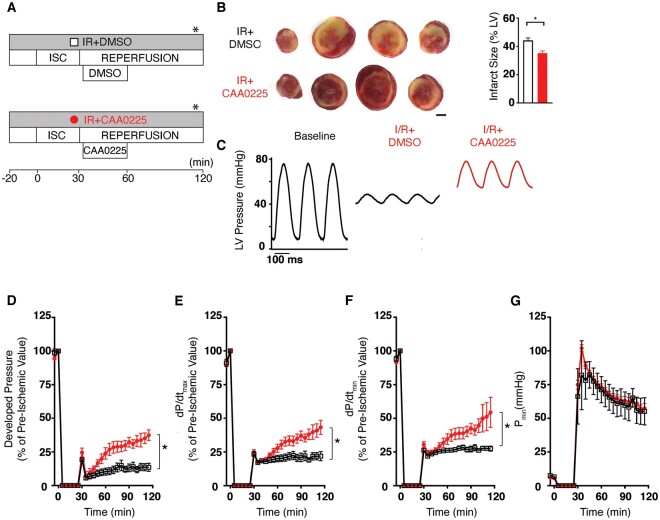
Cardiac function in *ex vivo* rat hearts during IR injury treated upon reperfusion with the cathepsin-L inhibitor CAA0225. (*A*) Schematic of the two protocols used. (*B*) Typical TTC staining of heart slices, where the red staining represents live tissue and the pale unstained colour is dead tissue (3 mm scale bar). The mean infarct size is shown on the right for IR+DMSO (*n* = 5) and IR+CAA0225 (*n* = 7). (*C*) Typical LV pressure measured at the time point shown in part A of this figure (*). (*D*–*G*) Mean LV pressure data for IR+DMSO (*n* = 5) and IR+CAA0225 (*n* = 7). (*D*) Developed LV pressure. (*E*) Maximum rate of rise (dP/dt_max_). (*F*) Maximum rate of fall (dP/dt_min_). (*G*) Minimum pressure (Pmin). *P <* *0.05. Data were assessed for normality and are expressed as mean ± standard error of the mean (SEM). Statistical comparisons were made by a two-sample Student’s *T*-test on the raw data. Multiple groups were compared with analysis of variance (ANOVA). A significance level of *P *<* *0.05 was considered significant. In experiments involving serial measurements on *ex vivo* hearts, the final measurement was taken as the relevant summary statistic in order to answer the research hypothesis of whether there would be changes in population mean values between treatment groups by the end of the experiment.

To assess therapeutic potential, CAA0225 was administered to Langendorff-perfused rat hearts early in reperfusion (a therapeutic window for patients undergoing PPCI that also coincides with cathepsin-L release; *Figure 5A*) in a randomized and blinded study. CAA0225 applied during reperfusion decreased infarct size to 80% of that in the IR+DMSO (*Figure 5B*; *P *<* *0.05). Furthermore, CAA0225 improved overall LV developed pressure, dP/dt_max_ and dP/dt_min_ at 120 min post-reperfusion to 276%, 198%, and 198%, respectively, of that in the IR+DMSO group (*Figure 5C*–*F*) with no statistically significant change in P_min_ (*Figure 5G*).

In separate experiments, we confirmed our findings using a different highly selective cathepsin-L inhibitor called Cathepsin Inhibitor IV. Unlike CA00225, Cathespin Inhibitor IV is a potent reversible inhibitor of cathepsin-L. Cathepsin Inhibitor IV applied for 20 min during reperfusion (Supplementary material online, *Figure* *S3A*) improved overall LV developed pressure, dP/dt_max_ and dP/dt_min_ post-drug application [to 175%, 178% (*P* < 0.05) and 151% (*P* = 0.05), respectively], of that in the IR+DMSO group (Supplementary material online, *Figure* *S3B*–*G*).

### 3.6 Cathepsin-L activity in *ex vivo* rat hearts

**Figure 6:**
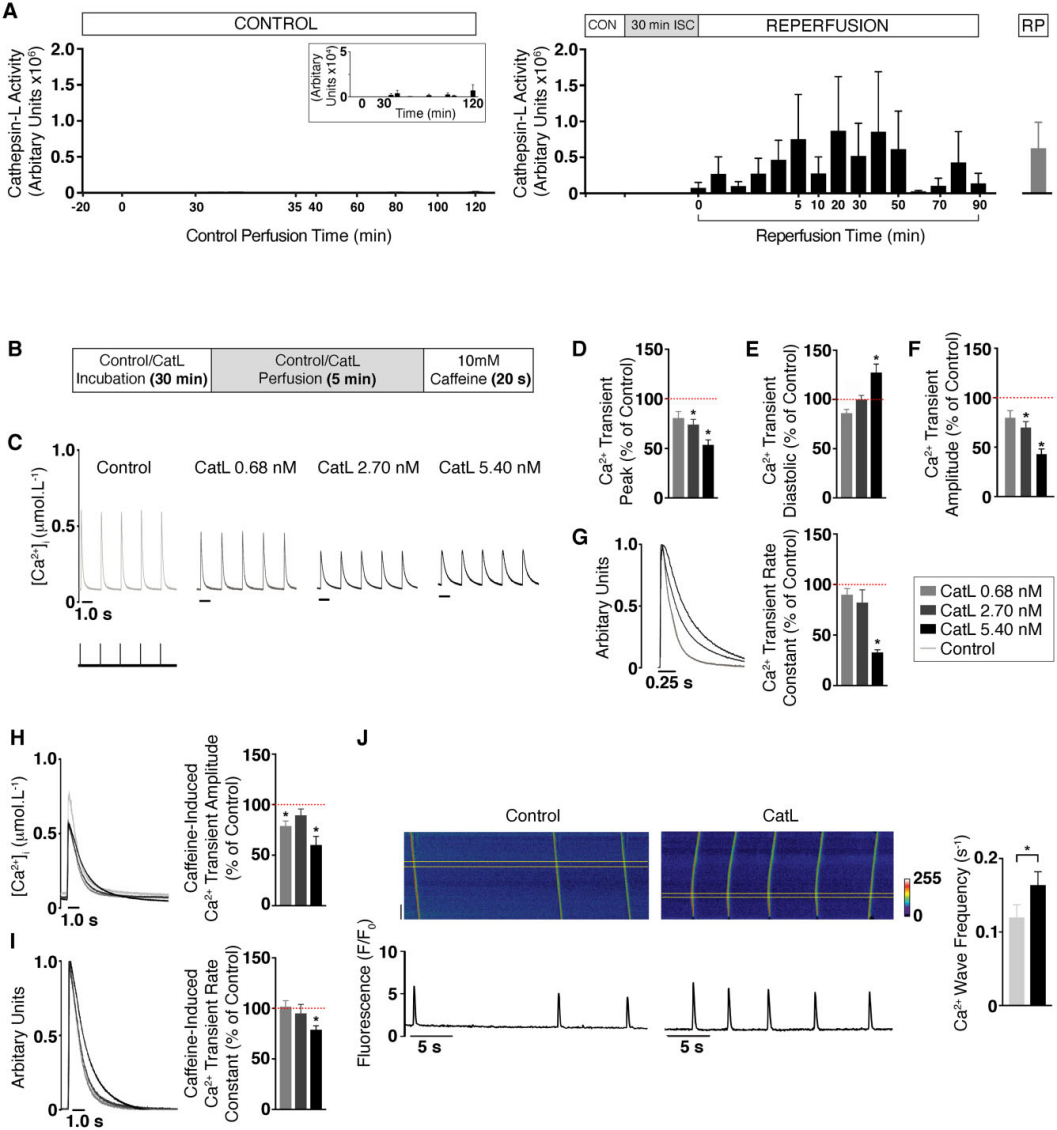
Extracellular cathepsin-L and rat cardiomyocyte function. (*A*) Cathepsin-L activity in coronary effluent samples from control perfused hearts (*n* = 3; left) and hearts undergoing myocardial-reperfusion injury (*n* = 3; right). The insert shows high resolution scale (1 × 10^4^). Recombinant mouse cathepsin-L (RP CatL; 0.68 nmol.L^−1^) was the assay control. (*B*) Schematic of the protocol for isolated cardiomyocyte experiments (control group represents the vehicle). (*C*) Typical calcium transients with various concentrations of RP CatL. (*D*–*G*) Mean calcium transient parameters at RP CatL concentrations of 0.68 nmol.L^−1^ [*n* = 23 cardiomyocytes (4 hearts)], 2.70 nmol.L^−1^ [*n* = 14 (4)] and 5.40 nmol.L^−1^ [*n* = 18 (4)]. (*D*) Peak. (*E*) Diastolic. (*F*) Amplitude. (*G*) Normalized typical calcium transients indicate differing rates of decay (left) with mean calcium transient rate constant (right). (*H*) Typical caffeine-induced calcium transients with RP CatL (left) with mean amplitudes (right). (*I*) Normalized typical caffeine-induced calcium transients with RP CatL (left) with mean caffeine-induced calcium rate constants of decay (right). (*J*) Typical confocal line-scan images showing calcium waves (top) and averaged signals (bottom) of isolated cardiomyocytes perfused with control [left; *n* = 73 (6)] and RP [right; *n* = 71 (6)]. The mean calcium wave frequency is also shown (far right; *P *<* *0.05). The dotted red lines indicate 100%. Data are expressed as mean ± standard error of the mean (SEM). Statistical comparisons were made by a two-sample Student’s *T*-test on the raw data. In experiments where multiple isolated cardiomyocyte observations were obtained from each heart, the average cardiomyocyte data from each heart was used to determine differences between groups.

To corroborate the findings observed among STEMI patients regarding direct cardiac release of cathepsin-L during reperfusion injury, rat hearts were Langendorff-perfused and cathepsin-L activity measured in the coronary effluent. Control hearts had minimal detectable cathepsin-L activity whereas the reperfusion injury group exhibited increased cathepsin-L activity (*Figure 6A*). Cathepsin-L levels peaked at 20 min after the start of reperfusion, which was equivalent to the time when cathepsin-L was measured among patients post-PPCI. Recombinant cathepsin-L protein (RP) provided a positive control, with 0.68 nmol.L^−1^ giving an equivalent cathepsin-L activity level as that observed upon reperfusion injury (*Figure 6A*).

### 3.7 The effects of recombinant cathepsin-L on Ca^2+^ handling in adult rat cardiomyocytes

**Figure 7:**
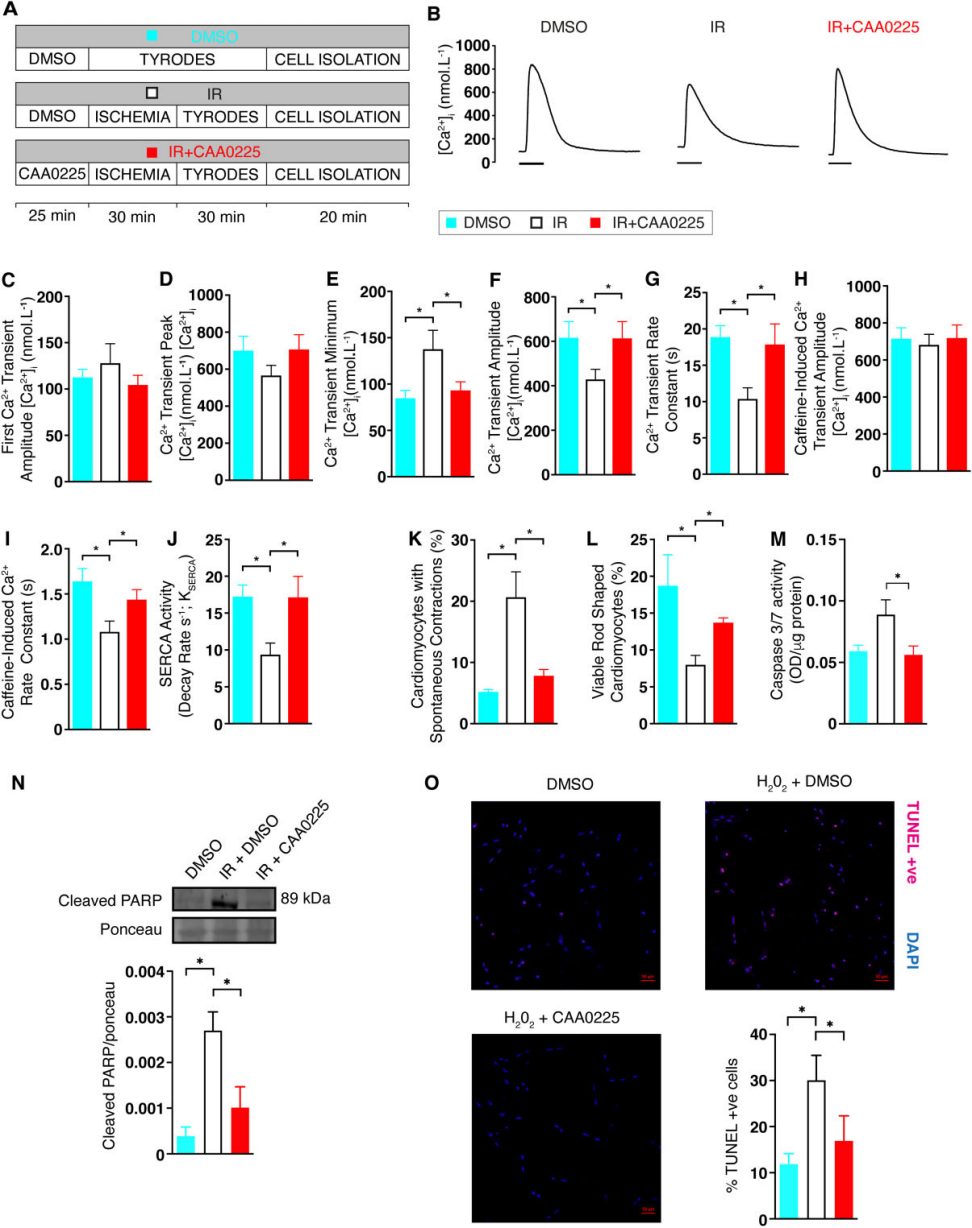
Effect of CAA0225 on calcium handling in rat cardiomyocytes with reperfusion injury. (*A*) Schematic of the three protocols used. (*B*) Typical calcium transient traces for the three protocols (100 ms scale bar). (*C*) Mean first calcium transient amplitude post-caffeine for DMSO [*n* = 12 cardiomyocytes (3 hearts)], IR+DMSO [*n* = 16 (3)] and IR+CAA0225 [*n* = 18 (3)]. (*D*) Steady state calcium transient peak calcium concentration. (*E*) Calcium transient minimum calcium concentration. (*F*) Calcium transient amplitude. (*G*) Calcium transient rate constant. (*H*) SERCA activity. (*I*) Caffeine-induced calcium release amplitude. (*K*) Percentage of cardiomyocytes with calcium waves in populations of unstimulated cardiomyocytes post-isolation for DMSO [*n* = 487 (5)], IR+DMSO [*n* = 330 (9)] and IR+CAA0225 [*n* = 535 (6)]. (*L*) Percentage of viable cardiomyocytes in populations of unstimulated cardiomyocytes post-isolation for DMSO [*n* = 792 (6)], IR+DMSO [*n* = 2001 (11)] and IR+CAA0225 [*n* = 957 (6)]. (*M*) Caspase 3/7 activity in isolated cardiomyocytes for DMSO (*N* = 5), IR+DMSO (*N* = 5), and IR+CAA0225 (*N* = 5). (*N*) Representative immunoblotting image for cleaved PARP-1 expression in isolated cardiomyocytes for DMSO (*N* = 3), IR + DMSO (*N* = 3), and IR+CAA0225 (*N* = 3). (*O*) Representative confocal microscopy images of TUNEL stained cardiomyocytes for DMSO (*N* = 6), H_2_O_2_ + DMSO (*N* = 6) and H_2_O_2_+CAA0225 (*N* = 6). Data are expressed as mean ± standard error of the mean (SEM). Statistical comparisons were made by a two-sample Student’s *T*-test on the raw data. A significance level of *P *<* *0.05 was considered significant. In experiments where multiple isolated cardiomyocyte observations were obtained from each heart, linear mixed modelling (SPSS Statistics v22) was used to determine differences between groups.

We next determined whether similar extracellular levels of cathepsin-L might alter sarcoplasmic reticulum (SR)-mediated calcium release and contractile function (*Figure 6B*). As shown in *Figures 6C and 7D*, the calcium transient peak (systolic [Ca^2+^]_i_) with increasing concentrations of cathepsin-L RP was 81%, 74%, and 54% of the respective control values. The calcium transient minimum (diastolic [Ca^2+^]_i_) with increasing concentrations of cathepsin-L RP was 86%, 100%, and 127% of the respective control values (*Figure 6C and E*). The changes in peak and minimum [Ca^2+^]_i_ with increasing concentrations of cathepsin-L RP resulted in a calcium transient amplitude that was 80%, 70%, and 43% of the respective controls (*Figure 6C and F*). Furthermore, the calcium transient rate constant with increasing concentrations of cathepsin-L RP was 90%, 82%, and 33% of the respective controls (*Figure 6G*).

We next determined whether the reduced calcium transient observed with cathepsin-L was related to a reduced SR calcium content. The caffeine-induced calcium transient amplitude (a measure of the SR calcium content) in response to increasing concentrations of cathepsin-L RP was 79%, 90%, and 60% of the respective control values (*Figure 6H*).

Sarco-endoplasmic reticulum calcium ATPase (SERCA)-mediated calcium uptake is bypassed during application of 10 mM caffeine and cytosolic calcium removal occurs predominantly via the sodium calcium exchanger (NCX). To assess whether the reduction in SR calcium content by cathepsin-L was related to calcium extrusion from the cell via altered NCX, the rate constant for decline of the caffeine-induced calcium transient was measured. The caffeine-induced calcium transient rate constant with increasing concentrations of cathepsin-L RP was 102%, 95%, and 79% of the respective control values (*Figure 6I*).

Spontaneous SR-mediated calcium release during diastole, which can result in calcium waves, is known to contribute to abnormal cardiomyocyte function including cell death. Using confocal microscopy, we established that cathepsin-L RP increased calcium wave frequency to 136% of the control levels (*Figure 6J*). Therefore, extracellular cathepsin-L can negatively affect both systolic and diastolic SR-mediated calcium release in cardiomyocytes.

### 3.8 Ca^2+^ handling in reperfused adult rat cardiomyocytes treated with CAA0225

To establish if inhibition of cathepsin-L using CAA0225 altered calcium handling post-reperfusion, rat hearts were treated as outlined in *Figure 7A*, with subsequent cardiomyocyte isolation and calcium handling measurements (*Figure 7B*).

Before electrical stimulation of cardiomyocytes, 10 mM caffeine was applied to empty the SR of calcium. The amplitude of the first electrically stimulated calcium transient post-caffeine is an index of calcium entry via the L-type calcium channel.^14^^,^^15^ This value was not altered by reperfusion in either the presence or absence of CAA0225 (*Figure 7C*). Following continuation of electrical stimulation, the steady state mean calcium transient peak was not appreciably changed (*Figure 7D*). In contrast, the minimum diastolic calcium concentration was increased by reperfusion to 163% of the control but normalized to control levels by CAA0225 (*Figure 7E*). These changes resulted in a reduced Ca^2+^ amplitude after reperfusion to 70% of the control value, which was restored to control levels by CAA0225 (*Figure 7F*). The Ca^2+^ transient rate constant of decay decreased with reperfusion to 55% of the control but was reversed by CAA0225 (*Figure 7G*). The SR Ca^2+^ content—determined by the amplitude of the caffeine-induced calcium transient at the end of the protocol—was not altered (*Figure 7H*). The NCX activity—determined by the caffeine-induced calcium transient rate constant—was decreased to 66% of the control value, an effect that was reversed by CAA0225 (*Figure 7I*). SERCA activity—assessed by the subtraction of the caffeine-induced calcium transient rate constant of decline (NCX activity; as SERCA is bypassed during application of 10 mM caffeine^16^) from the calcium transient rate constant of decline (which is mainly composed of both NCX and SERCA activity^16^)—was reduced to 54% of the control value and normalized by CAA0225 (*Figure 7J*). CAA0225 therefore had the ability to rectify abnormal systolic and diastolic calcium handling within cardiomyocytes and improve LV function post-reperfusion.

Increased diastolic calcium concentration and spontaneous release of calcium from the SR (a hall mark of which is calcium waves) has been linked to mechanisms that contribute to cell death post-reperfusion. We assessed whether inhibition of cathepsin-L using CAA0225 could alter the production of spontaneous contractile events that originate from calcium waves and cardiomyocyte cell death. Unstimulated (quiescent) populations of cardiomyocytes from hearts undergoing equivalent protocols to *Figure 7A* were used. The percentage of cardiomyocytes producing spontaneous contractile events during reperfusion increased to 397% of the control value, an effect that was prevented by CAA0025 (*Figure 7K*). The number of viable rod-shaped cardiomyocytes was decreased by reperfusion injury to 43% of the control value but restored by CAA0225 (*Figure 7L*). Apoptosis and activation of caspases can lead to cell death post-reperfusion. CAA0225 normalized the reperfusion-induced increase of caspase 3 and 7 activity (*Figure 7M*). PARP-1 [(ADP-ribose) polymerase-1] cleavage by caspase 3/7 is an established marker of apoptotic cell death.^17^^,^^18^ Immunoblotting experiments showed that CAA0225 normalized the increased cleavage of PARP-1 during reperfusion (*Figure 7N*). In separate experiments, the ability of CAA0225 to prevent apoptosis was assessed by TUNEL staining and hydrogen peroxide to induce apoptosis. CAA0225 prevented the hydrogen peroxide-induced increased in apoptosis (11.9 ± 2.3 vs. 30.0 ± 5.4 vs. 16.9 ± 5.4; control vs. hydrogen peroxide vs. hydrogen peroxide+CAA0225; *Figure 7O*).

## 4. Discussion

Here, using both clinical and integrative preclinical studies, we demonstrate that serum cathepsin-L is more than simply a consequence of a pathological process, but is directly released from the hearts of patients and is intrinsically involved in mediating cardiac dysfunction during reperfusion injury. Importantly, we also demonstrate that use of a highly specific, cell permeable, cathepsin-L inhibitor (CAA0225)^12^ can reduce infarct size and lead to improvement of systolic and diastolic cardiac function. Cathepsin-L therefore represents a therapeutic target with the potential to limit progression to heart failure among patients with STEMI.

Three major aspects regarding cathepsin-L were unknown among STEMI patients until now: whether cathepsin-L levels rise upon reperfusion; whether cardiac release of cathepsin-L occurs upon reperfusion; and whether cathepsin-L levels correlate with cardiac function post-reperfusion. We believe that the current study has addressed these three questions by taking the novel clinical approach of evaluating serum cathepsin-L, together with cardiac function using MRI, among patients undergoing PPCI. We demonstrated a remarkable similarity between our preclinical and clinical study in that cathepsin-L levels temporarily increased 20 min after reperfusion injury and were associated with a decreased cardiac contractile function (reduced stroke volume, cardiac index, and LVEF). Cardiac release of cathepsin-L upon reperfusion is the main source of increased cathepsin-L after reperfusion injury. Furthermore, patients with elevated serum cathepsin-L levels had increased infarct sizes. Surprisingly, cathepsin-L correlated with both cardiac function at 24 h post-PPCI and LVEF at 6 months. Although further clinical work is required, our data suggest that serum cathepsin-L levels might hold promise as a future biomarker for assessing patient outcome and optimizing drug therapy post-PPCI for STEMI.

Cathepsins are released from lysosomes into the cytosol and systemic circulation during pathological conditions.^19^^,^^20^ The degree to which this cathepsin release might alter cardiomyocyte function was unclear, particularly in the context of reperfusion injury. The current study found that both human and rat hearts are sources of extracellular cathepsin-L during myocardial ischaemia–reperfusion injury. The time profile of cathepsin-L release in isolated rat hearts demonstrated a gradual increase that peaked at 20 min following reperfusion (*Figure 6*). Importantly, release of cathepsin-L from human hearts was also substantially increased at 20 min following reperfusion induced by PPCI.

We next examined the effect of extracellular cathepsin-L on isolated cardiomyocyte function. Three concentrations of cathepsin-L were chosen. The first was the mean level released by the *ex vivo* hearts upon reperfusion, with the other two representing elevated levels (four-fold and eight-fold) observed in some hearts. These experiments demonstrated a dose-dependent reduction in the amplitude of SR calcium release (a surrogate for cardiomyocyte contraction), decreased SR calcium content and, at the two higher concentrations, a reduction of NCX and SERCA activity. Consequently, a raised diastolic calcium concentration in cardiomyocytes was observed, which is known to impair diastolic function of the heart.^21^ Cathepsin-L also increased the frequency of spontaneous calcium waves. Similar findings were reported when using extracellular application of cathepsin-L on cardiomyocytes.^15^ The subcellular pathways linking cathepsin-L and altered Ca^2+^ signalling are unknown. However, the novel data reported in this current study demonstrating that cathepsin-L can adversely affect calcium handling are important given the substantial evidence linking calcium dysregulation to mitochondrial failure, cardiac contractile dysfunction, and cell death during reperfusion injury and post-MI (cardiac remodelling).^22^ Collectively, our results demonstrate that extracellular cardiac cathepsin-L release might negatively alter cardiac function, leading to the hypothesis that inhibition of cathepsin-L could be beneficial in the context of reperfusion injury.

To test our hypothesis, we used CAA0225 in Langendorff-perfused hearts subjected to reperfusion injury. Pre-treatment of whole hearts with CAA0225 before induction of reperfusion injury reduced infarct size and almost doubled cardiac contractile function post-reperfusion, strongly suggesting that cathepsin-L inhibition is beneficial to cardiac function during reperfusion injury. We can rule out an indirect positive inotropic effect as CAA0225 did not alter diastolic and systolic parameters during a 25-min pretreatment of the hearts (data not shown). The simplest explanation for the improved cardiac function was prevention of cell death and increased survival of viable contractile myocardium. Nonetheless, it is also possible that preventing cathepsin-L activity upon entry into the cytoplasm and extracellular space (with subsequent paracrine effects) prevents cathepsin-L-mediated modification of SR-mediated calcium release and contractility of surviving myocardium, resulting in improved cardiac function. Although the current study was the first to demonstrate that CAA0225 can reduce infarct size and improve cardiac function post-reperfusion injury, previous studies in different organs have shown that inhibition of other cathepsins can also reduce cell death. Cathepsin-B inhibitors attenuate reperfusion injury-induced hepatocyte death^23^ and protect against neuronal death during global reperfusion injury in the brain.^24^

To investigate the potential of CAA0225 to improve calcium handling and limit cardiomyocyte death post-reperfusion injury, we treated hearts with this inhibitor and then isolated the cardiomyocytes post-reperfusion to examine calcium handling. We clearly showed that in the absence of CAA0225 reperfusion injury resulted in a reduction of calcium transient amplitude, reduced SERCA activity and reduced NCX activity (similar to the effect observed with the addition of cathepsin-L to healthy cardiomyocytes). The decreased ability of the reperfused cardiomyocyte to extrude calcium from the cell or for calcium to return to the SR as efficiently as healthy cardiomyocytes led to an accumulation of calcium within the cytosol (evidenced by a raised diastolic calcium concentration) and increased cardiomyocyte death (irreversible hypercontracture). Cardiac reperfusion injury is associated with an increase in cardiomyocyte activation of caspases and apoptosis, a form of programmed cell death, which contributes to adverse cardiac remodelling and reduced cardiac function.^25^^,^^26^ An increase in diastolic calcium concentration is known to lead to apoptosis and caspase activation^22^^,^^27–29^ Importantly, cathepsin K and B have been shown to induce cardiomyocyte apoptosis.^30–33^ However, the role that cathepsin L plays in cardiomyocyte apoptosis is less clear.^33–36^ We demonstrate that in the context of reperfusion injury CAA0225 completely normalized the increase in diastolic calcium handling post-reperfusion and led to a substantial reduction in apoptosis as measured using TUNEL staining, caspase 3/7 activity and PARP-1 cleavage. These data provide mechanistic insight regarding how targeting cathepsin-L reduces infarct size and improves cardiac function in *ex vivo* hearts post-reperfusion injury. Given that CAA0225 can penetrate the cell membrane and that the cardiomyocytes were continuously superfused, these cells had little, if any, opportunity to be exposed to external cathepsin-L, therefore demonstrating the importance of intracellular cathepsin-L for cardiac dysfunction post-reperfusion injury.

Despite the benefit afforded by the Langendorff preparation in demonstrating a cardiac-specific protective effect of CAA0225, it was important to establish whether CAA0225 was effective *in vivo* when introduced after induction of ischaemia but before reperfusion (i.e. in a potentially therapeutic window). Furthermore, other factors present *in vivo* could influence the efficacy of CAA0225, for example, inflammatory cells migrate to the heart post-reperfusion injury and contribute to tissue concentrations of cathepsin-L.^37^ We demonstrated that CAA0225 reduced infarct size and improved cardiac contractile function 4 weeks post-reperfusion, demonstrating that cathepsin-L inhibition *in vivo* has a sustained positive impact on cardiac function. Collectively, these results indicate that release of cathepsin-L is more than simply a bystander effect of cell death during IR injury, but rather intrinsically involved in the pathophysiology of cardiac dysfunction following IR injury.

The substantial benefits afforded by cathepsin-L inhibition using CAA0225 during reperfusion injury in the current study might appear counterintuitive given previous studies using cathepsin-L whole-body knockout mice with persistent cathepsin-L deficiency from birth. These mice exhibit ventricular enlargement, chamber dilation and impaired cardiac contraction,^38^ with reduced cardiac function when MI or cardiac hypertrophy are induced.^37^^,^^39^ However, our study differs from previous work in three ways. First, we investigated the role of cathepsin-L in the context of reperfusion injury. Second, acute inactivation of cathepsins by specific inhibitors cause only temporary deficiency and therefore can result in different effects from those seen using cathepsin-L knockout mice.^40^ Third, the knockout mice cannot release cathepsin-L into the serum upon injury. This latter point means that the knockout mice do not demonstrate what has been observed in separate studies involving several hundred patients; that is, an increased serum cathepsin-L associated with increasing severity of CHD, coronary artery stenosis, number of coronary artery branch luminal narrowings, and coronary collateral formation.^8–10^ These clinical observations, together with the findings of the current study, strongly suggest that whilst complete deficiency of cathepsin-L from birth is deleterious, excess cathepsin-L might also be harmful.

## 5. Conclusion

Preserving cardiac contractility, limiting infarct size and preventing maladaptive remodelling are key factors to limit progression from STEMI to heart failure. The present study has identified cathepsin-L as a potential therapeutic target. Additional basic and translational studies are now required to determine whether blocking cathepsin-L release or activity could mitigate disease progression among patients with MI, thereby improving survival rates, quality of life and the health-economic burden.

## Supplementary material

Supplementary material is available at *Cardiovascular Research* online.

## Authors’ contributions

Acquisition, analysis, or interpretation of data:

W.H., C.S.M.C., K.N., K.F., K.M., A.R., D.O.T., A.Z., D.C., D.C., M.M.Y.L., M.M.E., A.D., R.G., M.M.L., H.E., P.R., S.W., S.H., A.S., L.M.A., E.B.E., J.M.C., M.C.P., K.G.O., C.H., Oxford Acute Myocardial Infarction (OxAMI) Study, K.M.C., C.B.

Substantial contribution to the conception/design of the work/Drafted the work/Substantially revised it:

T.M., G.L.S., S.A.N., K.G.O., K.M.C., C.B., C.M.L.

**Conflict of interest:** none declared.

## Funding

Chief Scientist Office (CSO; ETM/263), ISSF and The Wellcome Trust (097821/Z/11/Z). W.H. was supported by the China Scholarship Council. K.M. (FS/15/54/31639) and D.C. (FS/12/28/29417) are supported by Fellowships from the British Heart Foundation (BHF). K.N. was supported by a PhD studentship from the BHF. The OxAMI study is supported by the Oxford BHF Centre of Research Excellence (RG/13/1/30181), BHF Chair Award (CH/16/1/32013), to K.M.C and by the National Institute for Health (NIHR) Oxford Biomedical Research Centre. D.T. was supported by a PhD studentship from the University of Glasgow School of Veterinary Medicine and AZ is funded by the Department of Laboratory Medicine, Faculty of Applied Medical Sciences, Albaha University, Al-Baha, Saudi Arabia and Saudi Arabian Cultural Bureau, London, UK. C.M.L is supported by a BHF programme grant (RG/20/6/35095).

## Data availability

The data sets generated during and/or analysed during the current study are available from the corresponding author on reasonable request.

## Supplementary Material

cvab204_Supplementary_Data

## Notes

Translational perspective New therapeutic targets are urgently required to limit myocardial damage after reperfusion injury. We identified cardiac release of the protease cathepsin-L among patients following primary percutaneous coronary intervention (PPCI). Elevated serum levels of cathepsin-L were associated with reduced contractile function and increased infarct size at 24 h and 6 months post-PPCI. Work conducted using animal models indicated that cardiac release of cathepsin-L mediated cardiac dysfunction following reperfusion injury. Specific inhibition of cathepsin-L prevented abnormal calcium handling, reduced infarct size and improved contractile function. These novel findings offer the prospect of targeting cathepsin-L-mediated cardiac dysfunction after PPCI.

## References

[1] Heusch G , Gersh BJ. The pathophysiology of acute myocardial infarction and strategies of protection beyond reperfusion: a continual challenge. Eur Heart J 2016;38:774–784. 10.1093/eurheartj/ehw224 27354052

[2] Hausenloy DJ , Yellon DM. Ischaemic conditioning and reperfusion injury. Nat Rev Cardiol 2016;13:193–209. 26843289 10.1038/nrcardio.2016.5

[3] Kehat I , Molkentin JD. Molecular pathways underlying cardiac remodeling during pathophysiological stimulation. Circulation 2010;122:2727–2735. 21173361 10.1161/CIRCULATIONAHA.110.942268 PMC3076218

[4] Heusch G , Libby P, Gersh B, Yellon D, Bohm M, Lopaschuk G, Opie L. Cardiovascular remodelling in coronary artery disease and heart failure. Lancet 2014;383:1933–1943. 24831770 10.1016/S0140-6736(14)60107-0 PMC4330973

[5] Roger VL. Epidemiology of heart failure. Circ Res 2013;113:646–659. 23989710 10.1161/CIRCRESAHA.113.300268 PMC3806290

[6] Hausenloy DJ , Botker HE, Engstrom T, Erlinge D, Heusch G, Ibanez B, Kloner RA, Ovize M, Yellon DM, Garcia-Dorado D. Targeting reperfusion injury in patients with ST-segment elevation myocardial infarction: trials and tribulations. Eur Heart J 2016;38:935–941. 10.1093/eurheartj/ehw145 PMC5381598 27118196

[7] Reiser J , Adair B, Reinheckel T. Specialized roles for cysteine cathepsins in health and disease. J Clin Invest 2010;120:3421–3431. 20921628 10.1172/JCI42918 PMC2947230

[8] Liu J , Sukhova GK, Yang JT, Sun JS, Ma LK, Ren A, Xu WH, Fu HX, Dolganov GM, Hu CC, Libby P, Shi GP. Cathepsin L expression and regulation in human abdominal aortic aneurysm, atherosclerosis, and vascular cells. Atherosclerosis 2006;184:302–311. 15982660 10.1016/j.atherosclerosis.2005.05.012

[9] Liu YX , Li XP, Peng DQ, Tan Z, Liu HM, Qing YN, Xue YQ, Shi GP. Usefulness of derum Cathepsin L as an independent biomarker in patients with coronary heart disease. Am J Cardiol 2009;103:476–481. 19195505 10.1016/j.amjcard.2008.10.011 PMC2752663

[10] Zhang J , Wang P, Huang YB, Li J, Zhu J, Luo X, Shi HM, Li Y. Plasma cathepsin L and its related pro/antiangiogenic factors play useful roles in predicting rich coronary collaterals in patients with coronary heart disease. J Int Med Res 2010;38:1389–1403. 20926012 10.1177/147323001003800421

[11] Kohlhauer M , Dawkins S, Costa ASH, Lee R, Young T, Pell VR, Choudhury RP, Banning AP, Kharbanda RK, Oxford Acute Myocardial Infarction S, Saeb-Parsy K, Murphy MP, Frezza C, Krieg T, Channon KM. Metabolomic profiling in acute ST-segment-elevation myocardial infarction identifies succinate as an early marker of human ischemia-reperfusion injury. J Am Heart Assoc 2018;7:e007546. 29626151 10.1161/JAHA.117.007546 PMC6015393

[12] Takahashi K , Ueno T, Tanida I, Minematsu-Ikeguchi N, Murata M, Kominami E. Characterization of CAA0225, a novel inhibitor specific for cathepsin L, as a probe for autophagic proteolysis. Biol Pharm Bull 2009;32:475–479. 19252298 10.1248/bpb.32.475

[13] McCarroll CS , He W, Foote K, Bradley A, McGlynn K, Vidler F, Nixon C, Nather K, Fattah C, Riddell AH, Bowman P, Elliott EB, Bell M, Hawksby C, MacKenzie SM, Morrison LJ, Terry A, Blyth K, Smith GL, McBride MW, Kubin T, Braun T, Nicklin SA, Cameron ER, Loughrey CM. Runx1 deficiency protects against adverse cardiac remodeling following myocardial infarction. Circulation 2018;137:57–70. 29030345 10.1161/CIRCULATIONAHA.117.028911 PMC5757664

[14] Trafford AW , Diaz ME, Negretti N, Eisner DA. Enhanced Ca^2+^ current and decreased Ca^2+^ efflux restore sarcoplasmic reticulum Ca^2+^ content after depletion. Circ Res 1997;81:477–484. 9314828 10.1161/01.res.81.4.477

[15] Elliott EB , McCarroll D, Hasumi H, Welsh CE, Panissidi A, Jones NG, Rossor C, Tait A, Smith GL, Mottram JC, Morrison LJ, Loughrey CM. *Trypanosoma* brucei cathepsin-L increases arrhythmogenic sarcoplasmic reticulum-mediated calcium release in rat cardiomyocytes. Cardiovasc Res 2013;100:325–335. 23892734 10.1093/cvr/cvt187 PMC3797627

[16] Bode EF , Briston SJ, Overend CL, O'Neill SC, Trafford AW, Eisner DA. Changes of SERCA activity have only modest effects on sarcoplasmic reticulum Ca^2+^ content in rat ventricular myocytes. J Physiol 2011;589:4723–4729. 21825024 10.1113/jphysiol.2011.211052 PMC3213419

[17] Gobeil S , Boucher CC, Nadeau D, Poirier GG. Characterization of the necrotic cleavage of poly(ADP-ribose) polymerase (PARP-1): implication of lysosomal proteases. Cell Death Differ 2001;8:588–594. 11536009 10.1038/sj.cdd.4400851

[18] Los M , Mozoluk M, Ferrari D, Stepczynska A, Stroh C, Renz A, Herceg Z, Wang ZQ, Schulze-Osthoff K. Activation and caspase-mediated inhibition of PARP: a molecular switch between fibroblast necrosis and apoptosis in death receptor signaling. Mol Biol Cell 2002;13:978–988. 11907276 10.1091/mbc.01-05-0272 PMC99613

[19] Qin Y , Shi GP. Cysteinyl cathepsins and mast cell proteases in the pathogenesis and therapeutics of cardiovascular diseases. Pharmacol Ther 2011;131:338–350. 21605595 10.1016/j.pharmthera.2011.04.010 PMC3134138

[20] Appelqvist H , Waster P, Kagedal K, Ollinger K. The lysosome: from waste bag to potential therapeutic target. J Mol Cell Biol 2013;5:214–226. 23918283 10.1093/jmcb/mjt022

[21] Bers DM. Cardiac sarcoplasmic reticulum calcium leak: basis and roles in cardiac dysfunction. Annu Rev Physiol 2014;76:107–127. 24245942 10.1146/annurev-physiol-020911-153308

[22] Garcia-Dorado D , Ruiz-Meana M, Inserte J, Rodriguez-Sinovas A, Piper HM. Calcium-mediated cell death during myocardial reperfusion. Cardiovasc Res 2012;94:168–180. 22499772 10.1093/cvr/cvs116

[23] Ben-Ari Z , Mor E, Azarov D, Sulkes J, Tor R, Cheporko Y, Hochhauser E, Pappo O. Cathepsin B inactivation attenuates the apoptotic injury induced by ischemia/reperfusion of mouse liver. Apoptosis 2005;10:1261–1269. 16215674 10.1007/s10495-005-2358-1

[24] Xu Y , Wang J, Song X, Wei R, He F, Peng G, Luo B. Protective mechanisms of CA074-me (other than cathepsin-B inhibition) against programmed necrosis induced by global cerebral ischemia/reperfusion injury in rats. Brain Res Bull 2016;120:97–105. 26562519 10.1016/j.brainresbull.2015.11.007

[25] Eefting F , Rensing B, Wigman J, Pannekoek WJ, Liu WM, Cramer MJ, Lips DJ, Doevendans PA. Role of apoptosis in reperfusion injury. Cardiovasc Res 2004;61:414–426. 14962473 10.1016/j.cardiores.2003.12.023

[26] Teringova E , Tousek P. Apoptosis in ischemic heart disease. J Transl Med 2017;15:87. 28460644 10.1186/s12967-017-1191-y PMC5412049

[27] Chen X , Zhang X, Kubo H, Harris DM, Mills GD, Moyer J, Berretta R, Potts ST, Marsh JD, Houser SR. Ca^2+^ influx-induced sarcoplasmic reticulum Ca^2+^ overload causes mitochondrial-dependent apoptosis in ventricular myocytes. Circ Res 2005;97:1009–1017. 16210547 10.1161/01.RES.0000189270.72915.D1

[28] Báthori G , Csordás G, Garcia-Perez C, Davies E, Hajnóczky G. Ca^2+^-dependent control of the permeability properties of the mitochondrial outer membrane and voltage-dependent anion-selective channel (VDAC). J Biol Chem 2006;281:17347–17358. 16597621 10.1074/jbc.M600906200

[29] Lu FH , Fu SB, Leng X, Zhang X, Dong S, Zhao YJ, Ren H, Li H, Zhong X, Xu CQ, Zhang WH. Role of the calcium-sensing receptor in cardiomyocyte apoptosis via the sarcoplasmic reticulum and mitochondrial death pathway in cardiac hypertrophy and heart failure. Cell Physiol Biochem 2013;31:728–743. 23711498 10.1159/000350091

[30] Hong BK , Kwon HM, Byun KH, Kim D, Choi EY, Kang TS, Kang SM, Chun KJ, Jang Y, Kim HS, Kim M. Apoptosis in dilated cardiomyopathy. Korean J Intern Med 2000;15:56–64. 10714093 10.3904/kjim.2000.15.1.56 PMC4531747

[31] Stoka V , Turk B, Schendel SL, Kim TH, Cirman T, Snipas SJ, Ellerby LM, Bredesen D, Freeze H, Abrahamson M, Bromme D, Krajewski S, Reed JC, Yin XM, Turk V, Salvesen GS. Lysosomal protease pathways to apoptosis. Cleavage of bid, not pro-caspases, is the most likely route. J Biol Chem 2001;276:3149–3157. 11073962 10.1074/jbc.M008944200

[32] Guo R , Hua Y, Ren J, Bornfeldt KE, Nair S. Cardiomyocyte-specific disruption of Cathepsin K protects against doxorubicin-induced cardiotoxicity. Cell Death Dis 2018;9:692. 29880809 10.1038/s41419-018-0727-2 PMC5992138

[33] O'Toole D , Zaeri AAI, Nicklin SA, French AT, Loughrey CM, Martin TP. Signalling pathways linking cysteine cathepsins to adverse cardiac remodelling. Cell Signal 2020;76:109770. 32891693 10.1016/j.cellsig.2020.109770

[34] Heinrich M , Neumeyer J, Jakob M, Hallas C, Tchikov V, Winoto-Morbach S, Wickel M, Schneider-Brachert W, Trauzold A, Hethke A, Schütze S. Cathepsin D links TNF-induced acid sphingomyelinase to Bid-mediated caspase-9 and -3 activation. Cell Death Differ 2004;11:550–563. 14739942 10.1038/sj.cdd.4401382

[35] Blomgran R , Zheng L, Stendahl O. Cathepsin-cleaved Bid promotes apoptosis in human neutrophils via oxidative stress-induced lysosomal membrane permeabilization. J Leukoc Biol 2007;81:1213–1223. 17264306 10.1189/jlb.0506359

[36] Hamid T , Guo SZ, Kingery JR, Xiang X, Dawn B, Prabhu SD. Cardiomyocyte NF-kappaB p65 promotes adverse remodelling, apoptosis, and endoplasmic reticulum stress in heart failure. Cardiovasc Res 2011;89:129–138. 20797985 10.1093/cvr/cvq274 PMC3002872

[37] Sun M , Chen M, Liu Y, Fukuoka M, Zhou K, Li G, Dawood F, Gramolini A, Liu PP. Cathepsin-L contributes to cardiac repair and remodelling post-infarction. Cardiovasc Res 2011;89:374–383. 21147810 10.1093/cvr/cvq328

[38] Stypmann J , Glaser K, Roth W, Tobin DJ, Petermann I, Matthias R, Monnig G, Haverkamp W, Breithardt G, Schmahl W, Peters C, Reinheckel T. Dilated cardiomyopathy in mice deficient for the lysosomal cysteine peptidase cathepsin L. Proc Natl Acad Sci USA 2002;99:6234–6239. 11972068 10.1073/pnas.092637699 PMC122932

[39] Sun M , Ouzounian M, de Couto G, Chen M, Yan R, Fukuoka M, Li G, Moon M, Liu Y, Gramolini A, Wells GJ, Liu PP. Cathepsin-L ameliorates cardiac hypertrophy through activation of the autophagy-lysosomal dependent protein processing pathways. J Am Heart Assoc 2013;2:e000191. 23608608 10.1161/JAHA.113.000191 PMC3647266

[40] Lankelma JM , Voorend DM, Barwari T, Koetsveld J, Van der Spek AH, De Porto AP, Van RG, Van Noorden CJ. Cathepsin L, target in cancer treatment? Life Sci 2010;86:225–233. 19958782 10.1016/j.lfs.2009.11.016

