# Supplementary material for: Inhibition of myocardial cathepsin-L release during reperfusion following myocardial infarction improves cardiac function and reduces infarct size
Source: Cardiovasc Res. 2021 Jun 16;118(6):1535–47. doi: 10.1093/cvr/cvab204 (PMC9074968; doi:10.1093/cvr/cvab204)
Supplement: cvab204_Supplementary_Data [file cvab204_supplementary_data.pdf]

SUPPLEMENTARY MATERIAL

Detailed Methods

Human study population

A prospective single-centre cohort study was performed between January 26, 2015 and September 19, 2016 in a regional cardiac centre. Research staff screened patients with acute STEMI undergoing emergency invasive management. Acute STEMI management followed current guidelines[1, 2]. Inclusion criteria were age ≥ 18 years; symptom onset of ≤ 6 hours; and an occluded culprit artery (i.e. TIMI flow grade 0/1) (Supplementary Material online, Tables S1 and S2). Exclusion criteria were inability to give informed consent; cardiogenic shock; and a contraindication to MRI (e.g. pacemaker, defibrillator, or glomerular filtration rate <30 ml/min/1.73 m^2)[3, 4]. If patients fulfilled the eligibility criteria at angiography, they were invited to participate in the present study. All participants (n=83) provided witnessed informed consent. The study was approved by the National Research Ethics Service (reference 14/WS/0085) and was publicly registered (https://www.hra.nhs.uk/planning-and-improving-research/application-summaries/research-summaries/cathepsins-in-stemi/).

Measurement of serum levels of cathepsin-L

Systemic blood samples were taken at pre-reperfusion of the occluded culprit coronary artery; post-reperfusion; 24 h post STEMI; and at 6 months. The samples were centrifuged at 2,400 g for 15 min to separate the serum from the cellular component. Serum levels of cathepsin-L were measured by enzyme-linked immunosorbent assay (ELISA) according to the manufacturer's recommended protocol (USCN Life Science Inc.) and normalised to the total protein (TP) content of the serum determined by bicinchoninic acid (BCA) assay.

Cardiac magnetic resonance imaging (CMR) Acquisition

CMR was performed at 1.5T (MAGNETOM Avanto, Siemens Healthcare, Erlangen, Germany) using a scanner located in the Radiology Department, with an anterior phased-array body coil (12-element) and a posterior phased-array spine coil (24-element) at 1 day and 6 months post-MI[5].

The protocol included cine CMR (balanced steady-state free precession), a T2-prepared investigational prototype (T2 map, work-in-progress sequence 448 variant tfi2d1_77, Siemens Healthcare)[6, 7] and delayed-enhancement phase-sensitive inversion-recovery pulse sequences[8]. The LV dimensions were assessed using b-SSFP cinematographic breath-hold sequences. The heart was imaged in multiple parallel short-axis planes 8-mm thick separated by 2 mm gaps, as well as in the two-chamber, three-chamber, and four-chamber long-axis views. Typical sequence parameters were TE 1.2 ms, TR 42.8 ms, flip angle 77º, field of view 340 x 270mm, and matrix size 256 x 187. T2 maps were acquired in short-axis slices covering the whole ventricle, using a T2-prepared (T2P) balanced steady state free precession sequence (work-in-progress sequence 488, Siemens Healthcare, Erlangen, Germany)[6]. Typical imaging parameters were bandwidth 930 Hz/pixel; flip angle 70°; T2 preparations: 0, 24, and 55 ms, respectively; matrix 126 x 192 pixels; spatial resolution 2.2 × 1.8 × 8.0mm; and slice thickness 8mm.

Late gadolinium enhancement images covering the entire LV were acquired 10–15 min after intravenous injection of 0.15mmol.kg^{-1} of gadoterate meglumine (Gd^{2+}-DOTA, Dotarem, Guebert S.A.) using segmented phase-sensitive inversion recovery (PSIR) turbo fast low-angle shot[8]. Typical imaging parameters were matrix 256 x 156; flip angle 25°; TE 3.36 ms; bandwidth 130 Hz/pixel; echo spacing 8.7 ms; and trigger pulse 2. The voxel size was 1.8 x 1.3 x 8 mm^3. Inversion times were individually adjusted to optimize nulling of apparently normal myocardium (typical values, 200–300 ms).

CMR analysis

Data sets were anonymized to ensure operators were blinded to all other data. The analysis was conducted in a core laboratory by two researchers with more than 3 years of CMR experience, using dedicated CMR software (Medis suite version 2.1; Medis solutions, Leiden, the Netherlands). All imaging analyses were reviewed by an expert observer with more than 10 years of CMR experience. All scan acquisitions were spatially co-registered.

Infarction was defined by the occurrence of myocardial late gadolinium enhancement revealed in orthogonal planes, including phase swap acquisitions as appropriate to rule out artefacts. The myocardial mass of late gadolinium (in grams) was assessed using the full width half maximum thresholding method[9] on late gadolinium images and the QMass software (Medis suite version 2.1) and expressed as percentage of LV mass.

The AAR was assessed using the 2SD thresholding method on T2 maps with the QMass software and expressed as mass (35.4 ± 2.1 g) and percentage of LV mass (40.5 ± 1.7%).

Cathepsin-L activity in coronary artery effluent and cardiac tissue post-reperfusion
Cathepsin-L activity was determined using established protocols[10]. Briefly, 50µL of coronary effluent was incubated for 30 min with assay buffer (50 $mmol.L^{-1}$ sodium acetate, 2 $mmol.L^{-1}$ ethylenediaminetetraacetic acid [EDTA] and 2 $mmol.L^{-1}$ dithiothreitol [DTT]). The sample was incubated with Z-Leu-Arg-AMC (R&D systems, Abingdon, UK) for 1 h at 37 °C and the cleaved product measured as fluorescence at 380 nm excitation and 460 nm emission in an EnVision multilabel plate reader (Perkin Elmer, Seer Green, UK).

The cathepsin-L activity of frozen rat left ventricular myocardial was determined using a cathepsin-L activity assay (Abcam 65306) according to manufacturer's instructions. Briefly, 50µl of homogenized tissue sample was incubated with 50µl of CL buffer, 1µl of DTT and 2µl of 10mM CL substrate Ac-FR-AFC. Samples were incubated at 37°C for 2 hr and the cleaved product measured as fluorescence at excitation/emission 400/505nm. Cathepsin-L activity in each sample was subsequently normalized to total protein.

Immunoblotting
Isolated cardiomyocytes were lysed in radioimmunoprecipitation assay (RIPA) buffer. The composition of the RIPA buffer (in mmol/L) was: Tris (20), NaCl (150), EDTA (5), EGTA (5), DTT (1) plus 1.0% Triton X-100 and 0.5% deoxycholate. Tissue samples were homogenised in RIPA buffer using an ultrasonic device. Protease inhibitors added (in mmol/L) were: Sodium pyrophosphate (2.5), β-glycerophosphate (1.0), Na3VO4 (1.0), PMSF (1.0), NaF (2.0), plus 10µg leupeptin, one tablet of a protease inhibitor cocktail (Complete Mini, Roche, Germany) and one tablet of phosphatase inhibitor (Phos-stop, Roche, Germany) per 10mL buffer. The buffer was adjusted to pH 7.4. Protein concentration was determined using the bicinchoninic acid assay. BSA was used to produce a standard curve. Samples were mixed with β-mercaptoethanol as the reducing agent and a loading dye before heating at 65℃ for 10min. Samples were loaded at a concentration of 40 µg per well into 4–12% Bis-tris gels (NuPAGE, Life Technologies, UK). Electrophoresis conditions were 75 V for 15min followed by 165V for 75min. Protein was transferred to 0.45µm pore size nitrocellulose membranes (Life technologies, UK). Membranes were incubated overnight at 4℃ with primary antibodies against: cleaved PARP (1:500; 94885, Cell Signalling). Secondary antibodies were donkey anti-mouse IRDye 800 CW and donkey anti-rabbit IRDye 680RD (1:10000; 926-32212 and 926-68073, Li-Cor). Membranes were washed and imaged on the LI-COR Odyssey CLx Imaging System (LI-COR Biosciences, Nebraska, USA). Images were optimised and analysed using the Image Studio Lite v5.2 software (LI-COR Biosciences, Nebraska, USA).

Inducing apoptosis in rat cardiomyocytes using H2O2
Isolated rat cardiomyocytes were incubated with 1µM DMSO/CAA0225 (60 min @ 37°C). Cells were then incubated with hydrogen peroxide (0.4mM) (60 min @ 37°C) to induce cellular apoptosis. Cells were fixed with 4% paraformaldehyde and stained using a TUNEL assay kit (AbCam ab66110) and DAPI in accordance with manufacturer's instructions. Images were obtained using a spinning disk confocal microscope (10X/63X oil magnification), and the % of TUNEL positive rod-shaped cardiomyocytes was quantified (10X). The exposure and black/white contrast was optimized and settings were kept the same for all experiments.

Caspase 3/7 activity in isolated rat cardiomyocytes

The caspase 3/7 activity in isolated cardiomyocyte cell lysates was determined using a caspase activity assay kit (Abcam 39401) according to manufacturer's instructions. Briefly, 50μl of cell lysate was incubated with 50μl of 2X reaction buffer, 5μl of DTT and 5μl of 4mM substrate DEVD-p-NA. Samples were incubated at 37°C for 2 hr and the optical density of the cleaved product was measured at 405nm. Caspase 3/7 activity in each samples was subsequently normalized to total protein.

Epifluorescent Ca^{2+} transient measurements

Adult rat cardiomyocytes were isolated using previously published methods[11]. Isolated cardiomyocytes were incubated with 5 μM of a Ca^{2+} sensitive fluorophore (Fura-2-AM; Biotium Inc., Hayward, CA, USA) for 10 min. Cells were then re-suspended in a modified Krebs–Henseleit solution ([$mmol.L^{-1}$] $CaCl_2$ (1.8) NaCl (120), KCl (5.4), HEPES (20), NaH_2PO_4 (0.52), $MgCl_26H_2O$ (3.5), taurine (20), creatine (10), glucose (11.1), pH 7.4, 37ºC with activated cathepsin-L or a matching volume of activation buffer as the vehicle control. Cardiomyocytes were incubated at room temperature for 30 min to facilitate enzyme activity and cells to de-esterify the Ca^{2+}-sensitive dye. Cardiomyocytes were field stimulated (0.5 Hz, 2.0 ms duration, voltage set to 1.5x threshold) for 5 min (Cell Microcontrols) and superfused with HEPES buffered solution ([$mmol.L^{-1}$] NaCl 140.0, KCl 4.0, $MgCl_2$ 1.0, HEPES 5.0, glucose 11.1, $CaCl_2$ 1.8. pH 7.4, 37°C) containing cathepsin-L or vehicle. The fluorescence ratio (340/380 nm excitation wavelength) was measured with a spinning wheel spectrophotometer (Cairn Research UK) at a sampling rate of 5 kHz. Sarcoplasmic reticulum (SR) Ca^{2+} content was determined by rapid application of a 10$mmol.L^{-1}$ bolus of caffeine.

Confocal imaging of spontaneous SR-mediated Ca^{2+} release

Cardiomyocytes were loaded with Fluo-3AM Ca^{2+}-sensitive fluorophore for 10 min followed by resuspension and incubation with 1.8 mmol.L-1 $[Ca^{2+}]_o$ HEPES buffered solution and 5.4 nmol.L-1 activated recombinant cathepsin-L or the equivalent volume of vehicle (control) for 30 min (HEPES buffered solution ([$mmol.L^{-1}$]: NaCl 140.0, KCl 4.0, $MgCl_2$ 1.0, HEPES 5.0, glucose 11.1, $CaCl_2$ 1.8. pH 7.4, 37°C). Confocal images were recorded using a LSM 510 confocal system (Zeiss, UK). Fluo-3AM was excited at 488 nm (Ar LASER) and measured at >515nm using the epifluorescence optics of an inverted microscope with a 63x/1.2 NA water-immersion objective lens. Fluorescence was acquired using line-scan mode at 3.07 $ms.line^{-1}$ (1 line = 512 pixels); pixel dimension was 0.27□m. The scanning LASER line was orientated parallel with the long axis of the cell and placed approximately equidistant between the outer edge of the cell and the nucleus/nuclei to ensure the nuclear area was not included in the scan line. Fluorescence data were expressed as the ratio of quiescent fluorescence (F/F_0).

Langendorff perfusion and global ischemia–reperfusion

Hearts isolated from adult male Wistar rats were Langendorff perfused with Tyrodes solution ([$mmol.L^{-1}$]: 116.0 NaCl, 20.0 $NaHCO_3$, 0.4 Na_2HPO_4, 5.0 KCl, 1.0 $MgSO_4$-7H_2O, 11.0 glucose, 1.8 $CaCl_2$, 95% O_2 and 5% CO_2, 37°C) at 10 $mL.min^{-1}$. Left intraventricular pressure was measured with a solid-state pressure catheter (Millar/Transonic) and cling-film balloon. After 25 min steady state and 25 min vehicle (DMSO) or 1□$mol.L^{-1}$ CAA0225 (Merck, Darmstadt, Germany), a 30-min period of no flow ischemia (ISC) was induced followed by 90 min reperfusion. Control hearts (no ISC) were perfused continuously. Peak left ventricular (LV) pressure (P_{max}), minimum LV pressure (P_{min}), LV developed pressure (P_{dev}) and the maximal rate of LV pressure rise/fall (dP/dt_{max}, dP/dt_{min}) were recorded using LabChart 7.0 (ADInstruments, Oxford, UK). The P_{min} before ischemia was 5-7mmHg.

Measurement of infarct size of Langendorff perfused hearts

Transversely sectioned heart slices (2.0 mm thickness) were incubated with triphenyltetrazolium chloride (TTC) dissolved at 1% in a phosphate buffer for 15 min at 37°C to detect viable tissue. Heart slices were fixed in 10% neutral buffered formalin (CellPath, Powys, UK) at 4°C and digitally photographed and converted into planar images using Adobe Photoshop (Adobe). Viable tissue (stained red) was quantified using Image J (National Institutes of Health, Bethesda, MD, USA). The percentage infarct size was calculated using the following formula: (viable pixels/non-viable pixels) × 100%. Mean infarct size was calculated from eight faces of the equivalent four slices of each heart.

In vivo model of reperfusion injury

Male C57Bl/6 mice (age 9–12wk) were anesthetized in an induction chamber with 4% isoflurane and 100% oxygen. The anesthetized mice were intubated and ventilated using a small animal respirator (Harvard Apparatus, Germany) with 1.5% isoflurane. Preoperative analgesia was administered though the intraperitoneal route (5 mg/kg^{-1} carprofen and 0.1 mg/kg^{-1} buprenorphine). The chest was opened via the bluntly dissected pectoral muscles following a skin incision. The heart was exposed via the intercostal space between the third and fourth ribs. Ischaemia–reperfusion injury (IR) was induced using a 9-0 nylon suture to temporarily tie the left anterior descending (LAD) coronary artery against a short section of polyethylene tubing (PE-10; outer diameter, 0.61mm) with a 7-0 polypropylene suture. A single 0.25 mg dose of CAA0225 was delivered with 125 µL of vehicle (normal saline plus 15% DMSO) via intravenous injection within 2 min after LAD ligation. Temporary LAD ligation was released following 45 min of ischaemia by removing the polyethylene tubing. The lungs were fully inflated by applying positive end-expiratory pressure and the chest was closed in layers. Mice were extubated after regaining spontaneous breathing. M-mode serial echocardiography (15MHz paediatric ultrasonographic probe; Acuson Sequoia 512, Siemens UK) and pressure–volume loop measurements (ADVantage Pressure-Volume System; ADV500, Transonic; 1.2F 4.5mm spaced catheter via the right common carotid artery) was performed among mice undergoing coronary artery ligation using 0.5–1.0% isoflurane in 1.0 L.min^{-1} oxygen as previously described[11]. Area at risk (AAR) and infarct size were measured at 3 h post-reperfusion according to previously published protocols using 1% Evans blue (area not at risk), TTC (viable tissue) and white (infarct)[12]. Photographs of the heart slices were taken and the number of pixels counted by contouring the differentially coloured LV (five to eight parallel short axis slices per heart) using Adobe Photoshop. Percentage infarct of the AAR was corrected to slice weight.

Ischaemia–reperfusion cardiomyocyte experiments

Cardiomyocyte isolation was preceded by whole heart Langendorff perfusion with 25 min vehicle (0.06% DMSO) or 1.2 µmol.l^{-1} CAA0225 followed by 30 min of global ischemia and 30 min reperfusion. Quantification of viable cardiomyocytes (rod shaped) was performed using a haemocytometer (Neubauer). Assessment of spontaneous contractile activity was performed in Tyrodes solution using light microscopy (Leitz Wetzlar, Milton Keynes, UK) at x10 magnification, enabling observation of eight to 10 cardiomyocytes per field. Individual cardiomyocytes exhibiting at least one spontaneous contractile event within 60 s were noted and expressed as a percentage of the total cardiomyocyte number. The process was repeated in 10 separate fields.

Statistical analysis

Data were assessed for normality and are expressed as mean±standard error of the mean (SEM). Statistical comparisons were made by a two-sample Student's T-test on the raw data. Multiple groups were compared with analysis of variance (ANOVA). A significance level of $P<0.05$ was considered significant. In experiments involving serial measurements on ex-vivo hearts e.g. Figure 5 & 6, the final measurement was taken as the relevant summary statistic in order to answer the research hypothesis of whether there would be changes in population mean values between treatment groups by the end of the experiment[13]. In Figure 3, the data was analysed using analysis between groups at a particular time point (where significance is shown it depicts difference between groups; not from Time 0). In experiments where multiple isolated cardiomyocyte observations were obtained from each heart, linear mixed modeling (SPSS Statistics v22) or average data of cardiomyocytes from each heart was used to determine differences between groups[14]. N values are reported in the figure legends as n=cells (n=hearts). Pearson correlation was used to investigate the association between $\log_{10}$ cathepsin levels and subsequent MRI parameters in patients undergoing PPCI.

For the human population study:
Our primary objective was to characterise cathepsin levels in the blood after acute ST elevation myocardial infarction and determine the relationships between cathepsin-L concentrations and heart injury (infarct burden) at 24 hours and six months post-MI, i.e. the correlation between the area-under-the curve (AUC) for cathepsin-L concentration and final infarct size. Sample size calculations predicted that at least 38 measurements would allow a minimal clinically significant correlation between cathepsin AUC and final infarct size of 0.5 or more to be detected with 90% power and 5% significance. A sample size of 50 would allow for approximately 25% dropout. These data enabled an estimate of the standard deviation of cathepsin-L concentrations which may be useful to inform future clinical studies.

Limitations

Patients not suitable for MRI with gadolinium contrast were excluded (pacemaker, defibrillator, or glomerular filtration rate <30 ml/min/1.73 m^2). ST elevation MI with reduced flow down the culprit artery were enrolled in this study, and these findings are not generalizable to all STEMI presentations, or to non-ST elevation MI.

References

1. O'Gara PT, Kushner FG, Ascheim DD, Casey DE, Jr., Chung MK, de Lemos JA, Ettinger SM, Fang JC, Fesmire FM, Franklin BA, Granger CB, Krumholz HM, Linderbaum JA, Morrow DA, Newby LK, Ornato JP, Ou N, Radford MJ, Tamis-Holland JE, Tommaso JE, Tracy CM, Woo YJ, Zhao DX and Force CAT. 2013 ACCF/AHA guideline for the management of ST-elevation myocardial infarction: executive summary: a report of the American College of Cardiology Foundation/American Heart Association Task Force on Practice Guidelines. *Circulation*. 2013;127:529-55.

2. Levine GN, Bates ER, Bittl JA, Brindis RG, Fihn SD, Fleisher LA, Granger CB, Lange RA, Mack MJ, Mauri L, Mehran R, Mukherjee D, Newby LK, O'Gara PT, Sabatine MS, Smith PK and Smith SC, Jr. 2016 ACC/AHA Guideline Focused Update on Duration of Dual Antiplatelet Therapy in Patients With Coronary Artery Disease: A Report of the American College of Cardiology/American Heart Association Task Force on Clinical Practice Guidelines: An Update of the 2011 ACCF/AHA/SCAI Guideline for Percutaneous Coronary Intervention, 2011 ACCF/AHA Guideline for Coronary Artery Bypass Graft Surgery, 2012 ACC/AHA/ACP/AATS/PCNA/SCAI/STS Guideline for the Diagnosis and Management of Patients With Stable Ischemic Heart Disease, 2013 ACCF/AHA Guideline for the Management of ST-Elevation Myocardial Infarction, 2014 AHA/ACC Guideline for the Management of Patients With Non-ST-Elevation Acute Coronary Syndromes, and 2014 ACC/AHA Guideline on Perioperative Cardiovascular Evaluation and Management of Patients Undergoing Noncardiac Surgery. *Circulation*. 2016;134:e123-55.

3. Carrick D, Haig C, Ahmed N, McEntegart M, Petrie MC, Eteiba H, Hood S, Watkins S, Lindsay MM, Davie A, Mahrous A, Mordi I, Rauhalammi S, Sattar N, Welsh P, Radjenovic A, Ford I, Oldroyd KG and Berry C. Myocardial Hemorrhage After Acute Reperfused ST-Segment-Elevation Myocardial Infarction: Relation to Microvascular Obstruction and Prognostic Significance. *Circ Cardiovasc Imaging*. 2016;9:e004148.

4. Carrick D, Haig C, Rauhalammi S, Ahmed N, Mordi I, McEntegart M, Petrie MC, Eteiba H, Lindsay M, Watkins S, Hood S, Davie A, Mahrous A, Sattar N, Welsh P, Tzemos N, Radjenovic A, Ford I, Oldroyd KG and Berry C. Pathophysiology of LV Remodeling in Survivors of STEMI: Inflammation, Remote Myocardium, and Prognosis. *JACC Cardiovasc Imaging*. 2015;8:779-89.

5. Kramer CM, Barkhausen J, Flamm SD, Kim RJ, Nagel E and Society for Cardiovascular Magnetic Resonance Board of Trustees Task Force on Standardized P. Standardized cardiovascular magnetic resonance imaging (CMR) protocols, society for cardiovascular magnetic resonance: board of trustees task force on standardized protocols. *Journal of cardiovascular magnetic resonance : official journal of the Society for Cardiovascular Magnetic Resonance*. 2008;10:35.

6. Giri S, Chung YC, Merchant A, Mihai G, Rajagopalan S, Raman SV and Simonetti OP. T2 quantification for improved detection of myocardial edema. *Journal of cardiovascular magnetic resonance : official journal of the Society for Cardiovascular Magnetic Resonance*. 2009;11:56.

7. Verhaert D, Thavendiranathan P, Giri S, Mihai G, Rajagopalan S, Simonetti OP and Raman SV. Direct T2 quantification of myocardial edema in acute ischemic injury. *JACC Cardiovasc Imaging*. 2011;4:269-78.

8. Kellman P, Arai AE, McVeigh ER and Aletras AH. Phase-sensitive inversion recovery for detecting myocardial infarction using gadolinium-delayed hyperenhancement. *Magnetic resonance in medicine : official journal of the Society of Magnetic Resonance in Medicine / Society of Magnetic Resonance in Medicine*. 2002;47:372-83.

9. Khan JN, Nazir SA, Horsfield MA, Singh A, Kanagala P, Greenwood JP, Gershlick AH and McCann GP. Comparison of semi-automated methods to quantify infarct size and area at risk by cardiovascular magnetic resonance imaging at 1.5T and 3.0T field strengths. *BMC Res Notes*. 2015;8:52.

10. Annecke T, Chappell D, Chen C, Jacob M, Welsch U, Sommerhoff CP, Rehm M, Conzen PF and Becker BF. Sevoflurane preserves the endothelial glycocalyx against ischaemia-reperfusion injury. *BrJAnaesth*. 2010;104:414-421.

11. Fattah C, Nather K, McCarroll CS, Hortigon-Vinagre MP, Zamora V, Flores-Munoz M, McArthur L, Zentilin L, Giacca M, Touyz RM, Smith GL, Loughrey CM and Nicklin SA. Gene Therapy With Angiotensin-(1-9) Preserves Left Ventricular Systolic Function After Myocardial Infarction. *Journal of the American College of Cardiology*. 2016;68:2652-2666.

12. Bohl S, Medway DJ, Schulz-Menger J, Schneider JE, Neubauer S and Lygate CA. Refined approach for quantification of in vivo ischemia-reperfusion injury in the mouse heart. *AmJPhysiol Heart CircPhysiol*. 2009;297:H2054-H2058.

13. Matthews JN, Altman DG, Campbell MJ and Royston P. Analysis of serial measurements in medical research. *BMJ*. 1990;300:230-5.

14. Clarke JD, Caldwell JL, Horn MA, Bode EF, Richards MA, Hall MC, Graham HK, Briston SJ, Greensmith DJ, Eisner DA, Dibb KM and Trafford AW. Perturbed atrial calcium handling in an ovine model of heart failure: potential roles for reductions in the L-type calcium current. *Journal of molecular and cellular cardiology*. 2015;79:169-79.

Extended figure legend

Figure 1 Cathepsin-L levels among STEMI patients with divergent outcomes. The left-hand typical images **(A–D)** depict a patient who presented with chest pain of 45 min duration and a high level of cathepsin (area under the curve [AUC] measurement of 2.12 $ng.mg^{-1}$ total protein [TP] Log_{10}). **(A)** Pre-PPCI ECG performed by the paramedics depicting anterior ST elevation. **(B; Top)** Angiography, in this case the right anterior oblique–cranial view, depicting a culprit left anterior descending artery, TIMI flow 0 (yellow arrow). This is when the baseline (pre-PPCI) set of blood samples for cathepsin-L was taken from the radial artery sheath. **(B; Bottom)** Angiography post-reperfusion. Following intervention, the coronary artery was reperfused, with TIMI 3 flow restored (yellow arrows). A cardiac MRI scan was performed at day 1 post angiography **(C,D top)**. **(C top)** The area-at-risk was 51% LV mass on T2 parametric mapping, with remote myocardium depicted as blue, and ischemic myocardium depicted as grey (yellow arrows). The LV ejection fraction (LVEF) was 28.7% on the co-registered late enhancement image **(D top)**, with normal myocardium nulled to black, and acute scar depicted as hyperenhanced (white), with a central hypoenhanced region representing microvascular obstruction. The infarct size was 30.5 g (28% of LV mass). **(C,D bottom)** Depict T2 parametric map and the corresponding late enhancement image from the 6-month follow-up scan. No oedema was present on the T2 map **(C bottom)**. A reduction in infarct size to 11.6 g (11.2% of LV mass) was measured **(D bottom)**. The right-hand typical images **(E-H)** depict the progress of a participant with a low cathepsin level (1.84$ng.mg^{-1}$ TP Log_{10}) with anterior ST elevation on the pre-PPCI ECG **(E)**. On angiography, there was an acute left anterior descending artery occlusion **(F; top)** (right anterior oblique–cranial view), which was reperfused **(F; bottom)**. A cardiac MRI scan was performed at day 2 post angiography **(G,H)**. The area-at-risk was 46% LV mass on T2 parametric mapping **(G top)**, with remote myocardium depicted as blue, and ischemic myocardium depicted as grey (yellow arrows). The LV ejection fraction was 59.6% on the co-registered late enhancement image **(H top)**, with normal myocardium nulled to black, and acute scar depicted as hyperenhanced (white). The infarct size was smaller at 19.8 g (19% of LV mass). **(G,H bottom)** Depict T2 parametric map and the corresponding late enhancement image from the 6 month follow-up scan. No oedema was present on the T2 map **(G bottom).** A reduction in infarct size to 11.4 g (11% LV mass) was measured at follow-up **(H bottom)**. **(I)** Serum cathepsin-L levels among patients undergoing PPCI. **(J-L)** Statistically significant correlations (P<0.05) between MRI parameters at 24 h post-PPCI (black) and 6 months post-PPCI (red) and the AUC of cathepsin-L levels measure in the first 24 h post-MI. **(J)** LV ejection fraction. **(K)** Stroke volume indexed for body surface area (right). **(L)** Cardiac index. **(M)** Infarct size (see Figure S1 for *n* values for I–M).

Supplemental figure legends

Figure S1: CONSORT diagram. The schematic shows the number of patients assessed at each stage of the clinical study.

Figure S2: Mean cardiac function. This parameter was determined by cardiac magnetic resonance imaging (MRI) among patients at 24 h post-PPCI (n=62; grey) and 6 months post-PPCI (n=51; red). The measures assessed were **(A)** left ventricular (LV) ejection fraction; **(B)** stroke volume; **(C)** cardiac index; **(D)** LV end-systolic volume (LVESV); and **(E)** LV end-diastolic volume (LVEDV). Values are given as mean ± standard error of the mean. * $P<0.05$.

Figure S3: Cardiac function in ex vivo Langendorff perfused hearts during I/R with 1μM cathepsin L (CatL) Inhibitor IV. (A) Protocols used in ex vivo isolated hearts; (top) with DMSO vehicle, (bottom) with CatL IV inhibitor. **(B)** Mean data of developed LV pressure [IR+DMSO (black; *n*=7) and IR+CatL IV (red; *n*=6)]. **(C)** Area under the curve mean data of developed LV pressure for reperfusion period (70-85 min). **(D)** Mean data of dP/dT_{max} [IR+DMSO (black; *n*=7) and IR+CatL IV (red; *n*=6)]. **(E)** Area under the curve mean data of of dP/dT_{max} for reperfusion period (70-85 min). **(F)** Mean data of dP/dT_{min} [IR+DMSO (black; *n*=7) and IR+CatL IV (red; *n*=6)]. **(G)** Area under the curve mean data of dP/dT_{min} for reperfusion period (70-85 min). *$P<0.05$.

Supplemental table captions

Table S1: Demographic characteristics of the STEMI patients (n=60). Data (collected where possible) are given for those patients with both a 24 h cardiac magnetic resonance imaging scan and area under the curve measurements of cathepsin-L.

Table S2: Medications taken by the STEMI patients (n=60). Data are given for those patients with both a 24 h cardiac magnetic resonance imaging scan and area under the curve measurements of cathepsin-L at hospital admission and discharge.

Complete Gel for Figure 7N:

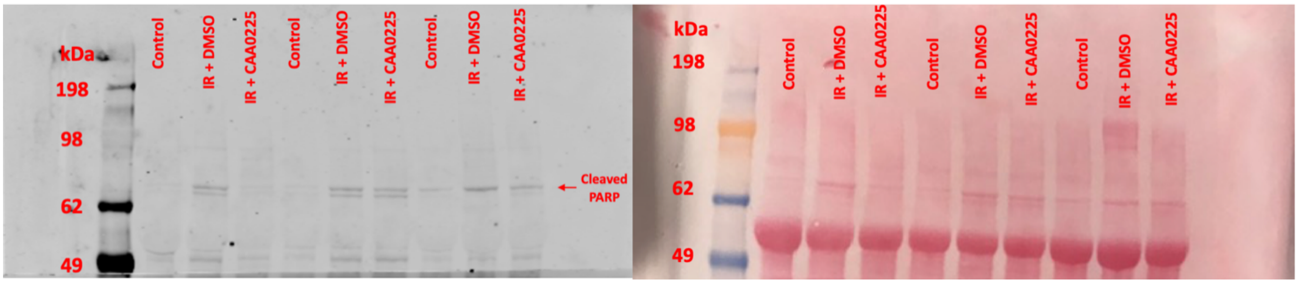

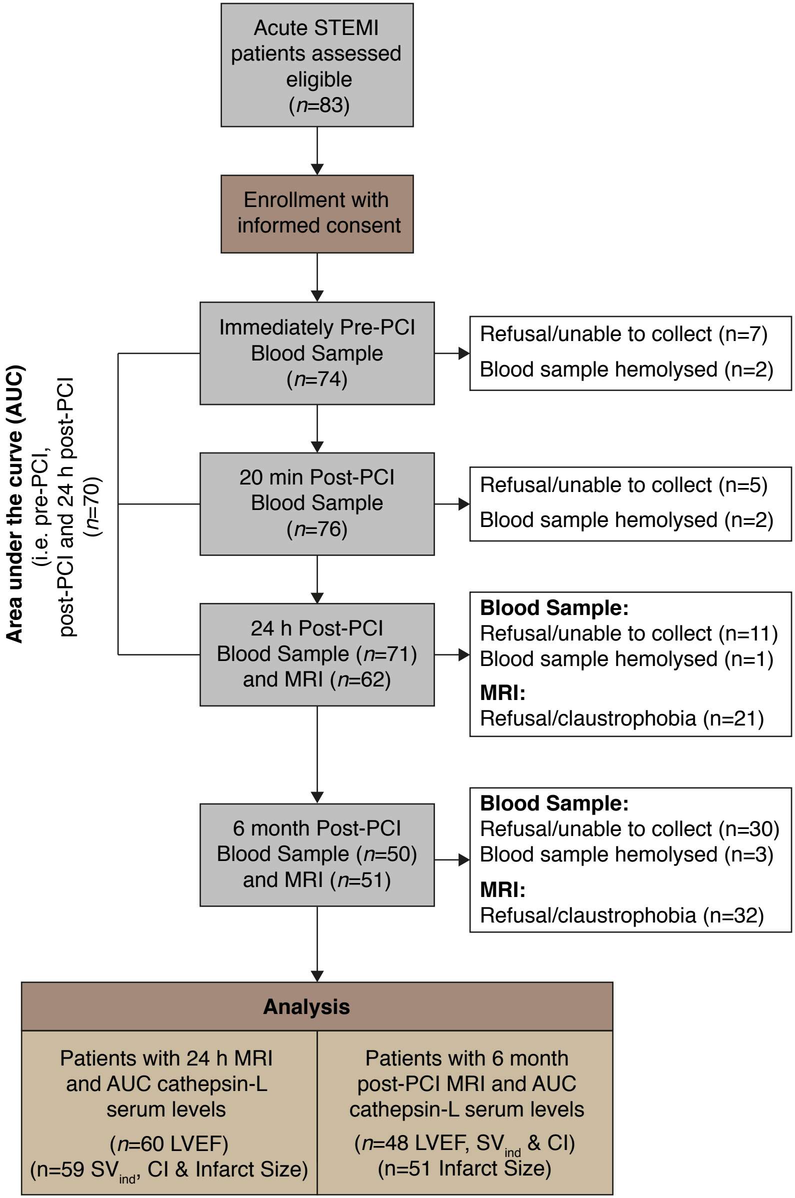

SUPPLEMENTARY FIGURE 1

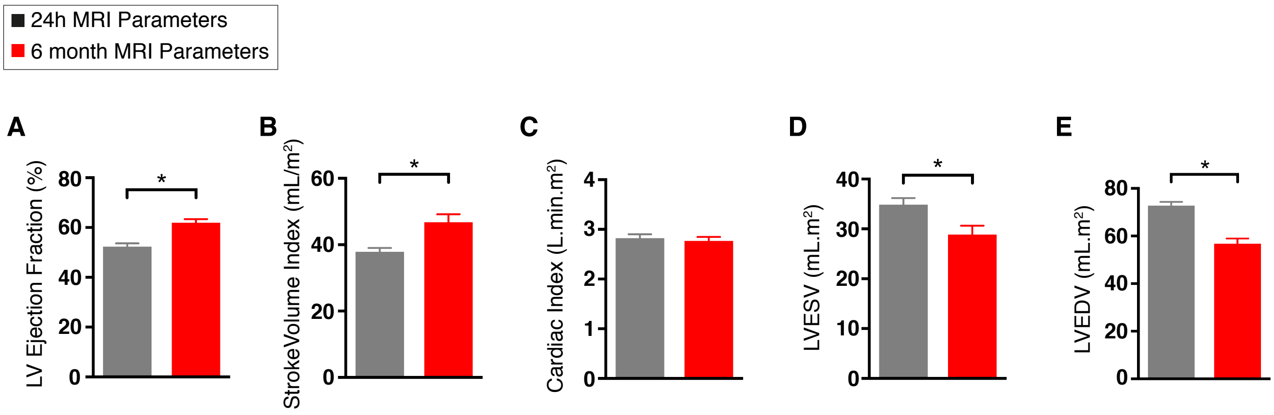

SUPPLEMENTARY FIGURE 2

SUPPLEMENTARY FIGURE 3

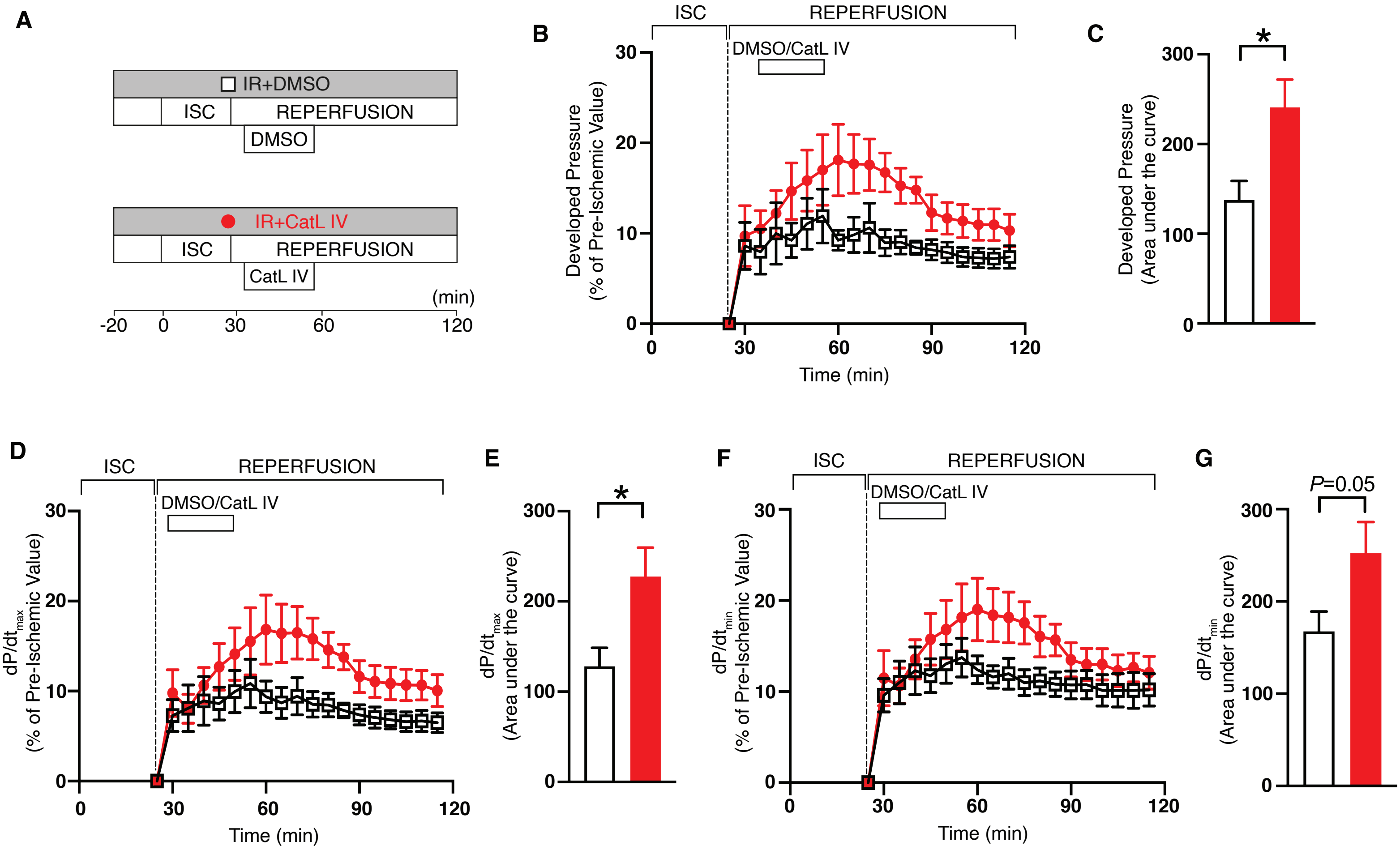

Table 1. Demographics of the Cathepsin-L STEMI participants.

Characteristics*	Patient *n* data collected from	Patients
Age, years	60	59.3 ± 10.3
Male sex, n (%)	60	47 (78.3)
Female sex, n (%)	60	13 (21.7)
BMI, (kg/m^2)	60	29.0 ± 5.4
Hypertension, n (%)	58	22 (37.9)
Current smoking, n (%)	57	24 (42.1)
Previous smoking, n (%)	59	14 (25.4)
Alcohol excess, n (%)	58	2 (3.5)
Hypercholesterolemia, n (%)	58	12 (20.7)
Diabetes mellitus†, n (%)	58	6 (13.8)
Previous angina, n (%)	58	1 (1.7)
Previous myocardial infarction, n (%)	59	5 (8.5)
Previous PCI, n (%)	58	5 (8.6)
Presenting characteristics		
Systolic blood pressure, mmHg	59	129.3 ± 29.3
Diastolic blood pressure, mmHg	59	74.3 ± 14.4
Time from symptom onset to reperfusion, min	60	119.6 ± 59.1
STEMI		
ST segment elevation, n (%)		
Anterior	58	20 (34.5)
Inferior	58	31 (53.4)
Lateral	58	1 (1.7)

Anterolateral	58	3 (5.2)
Inferolateral	58	1 (1.7)
Inferoposterior	58	2 (3.4)
Culprit artery, n (%)		
Left anterior descending	58	26 (44.8)
Left circumflex	58	5 (8.6)
Right coronary	58	27 (46.6)

*Values are mean ± SD, n (%).

†Diabetes mellitus was defined as a history of diet-controlled or treated diabetes.

BMI- body mass index, PCI- percutaneous coronary intervention, STEMI- ST-segment elevation myocardial infarction.

Table 2. Medications of the Cathepsin-L STEMI participants.

Medications*	Patient *n* data collected from	Patients
Admission		
Aspirin	60	7 (11.7)
Clopidogrel/Ticagrelor	60	3 (5.0)
Beta-blocker	60	10 (16.7)
ACE-I/ARB	60	18 (33.3)
Statin	60	22 (36.7)
Insulin	60	1 (1.7)
OHA	60	8 (15.0)
MRA	60	0 (0)
Discharge		
Aspirin	60	60 (100)
Clopidogrel/Ticagrelor**	60	60 (100)
Beta-blocker	60	59 (98.3)
ACE-I/ARB	60	59 (98.3)
Statin	60	60 (100.0)
Insulin	60	1 (1.7)
OHA	60	6 (10.0)
MRA	60	4 (6.7)

*Values are mean ± SD, n (%).

** 4 patients were discharged on clopidgrel due to contra-indications to ticagrelor

ACE-I = Angiotensin converting enzyme inhibitor, ARB = Angiotensin receptor blocker, OHA = Oral hypoglycaemic agent, MRA = Mineralocorticoid receptor antagonist.
